# Homeobox B13 activates the hypoxia‐inducible factor 1 pathway through histone lactylation thereby reprogramming lipid metabolism and promoting sorafenib resistance in hepatocellular carcinoma

**DOI:** 10.1002/ccs3.70077

**Published:** 2026-05-06

**Authors:** Qingqing Xie, Fangxia Teng, Ting Ding, Huaizhe Zhang, Jian Huang, Shu Zhang

**Affiliations:** ^1^ Center for Clinical Laboratories The Affiliated Hospital of Guizhou Medical University Guiyang China; ^2^ Department of Basic Clinical Laboratory Medicine School of Clinical Laboratory Science Guizhou Medical University Guiyang China

**Keywords:** H3K18la, hepatocellular carcinoma sorafenib resistance, HIF‐1 signaling pathway, histone Kla, HOXB13, lipid metabolism reprogramming

## Abstract

Resistance of hepatocellular carcinoma (HCC) to sorafenib represents a major clinical challenge, involving complex metabolic alterations and epigenetic regulatory changes. However, the underlying mechanisms remain incompletely understood. In this study, we found that the level of histone H3 lysine 18 lactylation (H3K18la), derived from lactate, was significantly elevated in sorafenib‐resistant HCC cells. Mechanistically, using Micrococcal Nuclease‐Chromatin Immunoprecipitation‐quantitative Polymerase Chain Reaction and related techniques, we demonstrated that H3K18la is directly enriched at the promoter region of homeobox B13 (HOXB13) and functions as a potent transcriptional activator to upregulate its expression. Further mechanistic investigations revealed that HOXB13 stabilizes hypoxia‐inducible factor‐1α (HIF‐1α) protein expression, thereby activating the HIF‐1 signaling pathway, promoting lipid metabolism reprogramming, and enhancing lipid accumulation. Functional experiments demonstrated that the inhibition of H3K18la or knockdown of HOXB13 effectively reversed lipid accumulation and significantly increased cellular sensitivity to sorafenib. This study systematically delineates a signaling cascade (the H3K18la‐HOXB13‐HIF‐1 axis) spanning metabolites, epigenetic modifications, transcriptional regulation, and downstream metabolic phenotypes, thereby deepening our understanding of tumor drug resistance mechanisms and providing potential therapeutic targets for overcoming HCC resistance to sorafenib.

## Graphical abstract

*Lactylation‐mediated metabolism reprogramming in HCC sorafenib resistance*. In sorafenib‐resistant hepatocellular carcinoma (HCC) cells, lactate accumulation drives histone H3 lysine 18 lactylation (H3K18la). This modification is specifically enriched in the promoter region of homeobox B13 (HOXB13), activating HOXB13 transcription and upregulating its expression. HOXB13 strongly activates the classical HIF‐1 signaling pathway by stabilizing hypoxia‐inducible factor‐1α (HIF‐1α) expression, reprogramming lipid metabolism in HCC cells, and providing survival advantages and energy reserves for tumor cells, ultimately leading to sorafenib resistance.

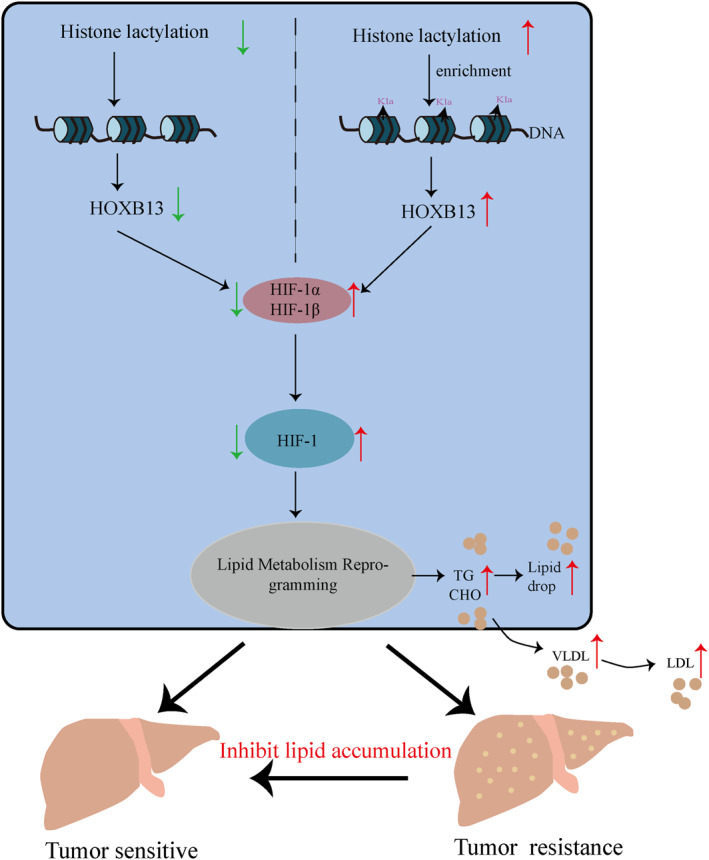

## 1 INTRODUCTION

Hepatocellular carcinoma (HCC) ranks among the most common malignant tumors. Its highly invasive nature and strong metastatic potential make it the most lethal subtype of liver cancer.^1^ For patients with early stage HCC, surgical resection, liver transplantation, and radiofrequency ablation are preferred treatment options that can improve patient survival rates in the short term. However, due to the insidious onset and nonspecific clinical manifestations of HCC, the majority of patients are diagnosed at an advanced stage, when conventional treatments are markedly less effective.^1^, ^2^ Sorafenib, approved by the FDA as the first‐line systemic therapy for advanced HCC,^3^ is a multitargeted kinase inhibitor. By suppressing the mitogen‐activated protein kinase pathway and vascular endothelial growth factor receptors, it inhibits tumor proliferation and angiogenesis,^4^, ^5^ hereby extending progression‐free survival in advanced patients. Despite its initial efficacy, most patients develop acquired resistance after prolonged treatment,^6^ which limits its therapeutic benefits. Therefore, elucidating the molecular mechanisms underlying sorafenib resistance is critical for improving future drug development and clinical applications.

Lysine lactylation (Kla) is a novel posttranslational modification (PTM) discovered in recent years, whose formation is closely related to lactate, a glycolytic metabolite.^7^ In the tumor microenvironment, the Warburg effect drives metabolic reprogramming in cancer cells,^8^ characterized by a strong dependence on glycolysis and the accumulation of large amounts of lactate. Elevated lactate levels provide abundant substrates for lactate‐mediated modifications, inducing histone lysine lactylation (Kla) and directly participating in the regulation of gene transcription.^7^ Several studies have shown that histone Kla serves as a critical link between cellular metabolic states and phenotypic outcomes, regulating diverse biological processes, including immune suppression, tumor drug resistance, and metabolic reprogramming.^9^, ^10^, ^11^ He et al.^12^ reported in prostate cancer and lung adenocarcinoma that defects in the Numb/Parkin pathway induce metabolic reprogramming, which subsequently leads to upregulation of histone Kla signaling near the transcription start site of the oncogene MYCN. This process promotes differentiation of tumor cells toward a neuroendocrine phenotype, resulting in reduced sensitivity to targeted therapies and the development of drug resistance. Sun et al.^13^ reported that histone Kla levels are elevated in colorectal cancer cells and enriched at the promoter regions of ABC transporters involved in drug efflux, thereby contributing to drug resistance by enhancing chemotherapeutic agent efflux. These findings indicate that Kla plays a crucial role in tumor drug resistance across multiple cancer types. However, its role in sorafenib resistance in HCC and the underlying molecular mechanisms remain unclear. Therefore, further investigation of this lactate‐dependent modification may provide novel therapeutic targets for overcoming sorafenib resistance in HCC.

Lipid metabolism reprogramming, a key component of metabolic dysregulation, plays a critical role in driving resistance to targeted therapies. As the central organ of lipid metabolism, the liver often exhibits enhanced fatty acid synthesis, increased lipid uptake, and extensive accumulation of lipid droplets during carcinogenesis. Intracellular lipid accumulation not only provides the structural basis for tumor cell membrane synthesis but also promotes resistance to therapeutic agents by activating anti‐apoptotic signaling pathways, increasing tolerance to oxidative stress, and inhibiting ferroptosis.^14^, ^15^, ^16^, ^17^ Mok et al.^18^ found that caspase‐3–mediated SREBP2 cleavage promotes cholesterol biosynthesis, thereby inducing resistance to sorafenib/lenvatinib through activation of the sonic hedgehog signaling pathway. Sun et al.^19^ reported that palmitoylation of PCSK9 enhances its interaction with PTEN, leading to lysosomal degradation of PTEN and consequently promoting sorafenib resistance in HCC via activation of the PI3K/AKT pathway and modulation of cholesterol metabolism. Hypoxia‐inducible factor‐1α (HIF‐1α) is a key transcription factor that mediates cellular responses to hypoxia. Its stability is markedly increased under hypoxic conditions; by transcriptionally activating genes involved in lipid transport and synthesis, it promotes extracellular lipid uptake and intracellular lipid biosynthesis, while simultaneously inhibiting lipid oxidation and degradation, ultimately resulting in excessive lipid accumulation. Du et al.^20^ demonstrated that HIF‐1 activation drives lipid deposition in clear cell renal cell carcinoma by suppressing fatty acid metabolism. Seo et al.^21^ showed that fatty acid–induced upregulation of FABP5 promotes lipid accumulation and cell proliferation in HCC via HIF‐1–driven lipid metabolic reprogramming. In addition, HIF‐1 contributes to sorafenib resistance by regulating multidrug resistance genes, angiogenesis, and epithelial–mesenchymal transition.^22^, ^23^, ^24^, ^25^, ^26^ However, the upstream epigenetic regulatory mechanisms governing HIF‐1–dependent lipid metabolic reprogramming in HCC resistance remain unclear.

Homeobox B13 (HOXB13) is a transcription factor that plays a critical spatiotemporal regulatory role in embryonic development, tissue differentiation, and organogenesis. Aberrant expression of HOXB13 is closely associated with the malignant progression of various tumors.^27^, ^28^ Zhan et al.^29^ demonstrated that co‐expression of HOXB13 and its target genes ABCG1, EZH2, and Slug promotes metastasis in lung adenocarcinoma and reduces sensitivity to cisplatin. In liver disease, increased HOXB13 expression has been associated with HCC progression, suggesting its potential as a prognostic biomarker.^30^ However, it remains unclear whether HOXB13 is involved in regulating lipid metabolism in HCC and the development of sorafenib resistance, whether it interacts with HIF‐1 in a synergistic or hierarchical regulatory manner, or whether HOXB13 transcription is regulated by epigenetic modifications such as histone Kla.

Based on the research background described above, we propose the following hypothesis: In sorafenib‐resistant HCC cells, aberrantly activated lactate metabolism promotes transcription and upregulates HOXB13 expression by inducing histone H3 lysine 18 lactylation (H3K18la). HOXB13 stabilizes HIF‐1α expression and facilitates its nuclear localization, thereby activating the HIF‐1 signaling pathway. This process drives lipid metabolism reprogramming, ultimately sustaining and enhancing sorafenib resistance. To verify this hypothesis, we aim to elucidate the relationship between Kla and sorafenib resistance through in vitro experiments, clarify the role of the H3K18la‐HOXB13‐HIF‐1 signaling axis in lipid metabolism reprogramming, and investigate potential strategies for restoring sorafenib sensitivity by targeting this axis.

## 2 MATERIALS AND METHOD

### 2.1 Cell lines

Huh7 cells were purchased from Servicebio (Wuhan, China), and Huh7 sorafenib–resistant cells (Huh7/SR) were purchased from Aoruicell (Shanghai, China). HCCLM3 and HCCLM3 sorafenib–resistant cells (HCCLM3/SR) were purchased from Meisen Chinese Tissue Culture Collections (Zhejiang, China). After cell recovery, expand and freeze the cells, and use them for recovery within 6 months. The Huh7 and HCCLM3 cell lines were cultured in a DMEM medium (Gibco, 11965092). The Huh7/SR cell line was cultured in a DMEM medium containing 2 μg/mL sorafenib, and the HCCLM3/SR cell line was cultured in a DMEM medium containing 10 μM sorafenib. All cell culture media were supplemented with 10% fetal bovine serum (Meisen CTCC, CTCC‐002‐071‐2) and 1% penicillin–streptomycin (Servicebio, G4003‐100ML). The cells were placed in a constant‐temperature incubator at 37°C and 5% CO2. Cells were passaged every 2–3 days; all cells used in the experiment were within 5–15 passages. All cell lines have been authenticated by STR DNA analysis and tested negative for *Mycoplasma* via PCR.

### 2.2 Western blot

Protein concentration was determined using the BCA protein assay kit (Solarbio, Beijing, China). Use SDS‐PAGE electrophoresis to separate the protein sample, and then transfer it to a PVDF membrane. Block the membrane with 5% skim milk; then, incubate it overnight with different primary antibodies at 4°C. Incubate the membrane with HRP‐conjugated goat anti‐mouse or goat anti‐rabbit secondary antibody at room temperature for 1 h. Western blot was processed using the Ecl chemiluminescence detection kit. For details on the antibodies used, see the Supplementary Methods.

### 2.3 Immunofluorescence

The adherent cell samples were washed twice with PBS, fixed at room temperature for 30 min with 4% paraformaldehyde, and washed three times with PBS. Permeate with 0.5% Triton X‐100 at room temperature for 30 min, wash with PBS three times, dry PBS with absorbent paper, and block with 5% BSA at room temperature for 60 min. Next, the cell samples were incubated with the specific primary antibody overnight at 4°C. On the second day, the cell samples were washed three times with PBST and incubated with the corresponding fluorescein conjugated secondary antibody at room temperature in the dark for 1 h. The cell nucleus was counterstained with DAPI antifluorescence quenching sealing agent (1 μg/mL) (Servicebio, Wuhan, China) for 5 min. Fluorescence images were captured and analyzed using ZEISS ZEN 3.9 software, with all image parameters (such as exposure time and laser intensity) kept consistent. For details regarding the antibodies used, see Supplementary Methods.

### 2.4 Cell cloning

Inoculate the cells into a 6‐well plate and culture continuously for 10–14 days, changing the fresh culture medium every 2–3 days. Wash the cells twice with PBS, and fix them at room temperature with 4% paraformaldehyde for 30 min. Stain with 0.1% crystal violet solution for 20 min. Wash the dye solution with PBS, take photos, and calculate the number of cell colonies.

### 2.5 Wound healing

Inoculate the cells into a 6‐well plate. When the cell density reaches 100%, use a pipette tip for scratch treatment and wash the cells with PBS to remove floating cells. Cells were cultured in a serum‐free medium, and photographs were taken within the following 24 h to monitor scratch closure. The results were analyzed using Image J analysis.

### 2.6 Micrococcal Nuclease‐Chromatin Immunoprecipitation‐quantitative Polymerase Chain Reaction

Micrococcal Nuclease‐Chromatin Immunoprecipitation‐quantitative Polymerase Chain Reaction (MNase ChIP‐qPCR) is performed using the gold standard ChIP protocol. Approximately, 5 × 10^7^ cells were cross‐linked with 1% formaldehyde at room temperature for 10 min. Add 0.125 M glycine solution, mix well, and incubate at room temperature for 5 min to terminate the cross‐linking. Wash the cells twice, and add a mixture of the cell nucleus extraction buffer (10 mM Tris HCl, pH 7.5, 4 mM MgCl_2_, 10 mM KCl, 0.5 mM TCEP, 0.4% NP‐40, 1 mM CaCl, protease inhibitor, etc.) for collection. After digesting the chromatin with 500 gel units of MNase at 37°C for 20 min, add EDTA to a final concentration of 1 M and SDS to 10% to stop the reaction. The resulting sample can be used for subsequent ChIP experiments. Take a portion of the obtained sample as 2% input, and divide the rest of the sample equally. Immunoprecipitation was performed overnight at 4°C using 5 μg of H3K18la antibody (PTM‐1427RM, PTM Bio) or 5 μg of IgG antibody (98136‐1‐RR, Proteintech). On the second day, use a magnetic rack to capture the magnetic bead‐antibody‐chromatin complexes, discard the supernatant, and wash the beads at 4°C. Add 100–150 μL of ChIP elution buffer to the washed complexes and input samples, and incubate with rotation at room temperature for 15 min. Separate the beads and complexes using a magnetic rack, and transfer the supernatant (containing eluted DNA) to a new PCR tube. Add NaCl to all supernatants and input samples to a final concentration of 200 mM. Reverse crosslinks overnight in a 65°C water bath. Subsequently, purify the DNA using the Tiangen DNA purification kit. The purified DNA can be used for qPCR.

Based on previously reported promoter active regions in the literature and our group's prior experimental findings, we designed three pairs of ChIP‐qPCR–specific primers covering the region from 2 kb upstream to 1 kb downstream of the HOXB13 transcription start site. This region contains multiple potential transcription factor binding sites and highly overlaps with histone modification enriched regions. The ChIP‐qPCR primer sequences (Servicebio, Wuhan, China) are shown in Table S1.

### 2.7 Reverse transcription quantitative polymerase chain reaction (RT‐qPCR)

Total cellular RNA was extracted using TRnaZol Reagent (NCM Biotech, M5101) according to the manufacturer's instructions. RNA concentration and purity were determined using a microspectrophotometer. One microgram of total RNA was used for reverse transcription to synthesize cDNA using RT Master Mix for qPCR II (gDNA digester plus) (MCE, HY‐K0511A). Perform quantitative PCR using TB Green Premix Ex Taq™ II (Tli RNaseH Plus) (Takara, RR820A). Analyze the final PCR results using the 2^(−ΔΔCt) algorithm. The RT‐qPCR primer sequences (Sangon, Shanghai, China) are shown in Table S2.

### 2.8 shRNA/OE sequence transfection

Knock down the expression of HOXB13 by targeting the short hairpin RNA (shHOXB13) sequence of HOXB13, and use nontargeted short hairpin RNA (shNC) as a negative control. Overexpress HOXB13 (OE HOXB13) and use an empty vector as a negative control. When the cell density reaches 70%–80%, remove the old medium, wash twice with PBS, and add 2 mL of Opti‐MEM medium per well (6‐well plate, serum‐free and antibiotic‐free). Prepare the transfection mixture and transfect the cells according to the instructions of Lipo8000^TM^ transfection reagent (Beyotime, Shanghai, China).

### 2.9 Lactate assay kit

Collect cells, and wash them twice with PBS. Centrifuge at 1000 rpm/min for 5 min to collect precipitated cells. Add 0.5 mL (0.1 M, pH 7.4) of the isotonic PBS buffer to the cell pellet, suspend the cells, sonicate (power 20%, run for 5 s, interval 15 s, and repeat for 4 min), centrifuge at 4000 rpm/min for 10 min, and take the homogenate supernatant for testing. Measure the concentration of intracellular lactate using a reagent kit (Nanjing Jiangcheng Bioengineering Institute, China). Use the BCA assay kit to detect protein concentration.

### 2.10 Cholesterol (CHO) and triglycoride (TG) testing

Prepare cell suspension at 1000 rpm/min, centrifuge for 10 min, and discard the supernatant. Wash the precipitate twice with PBS at 1000 rpm, centrifuge for 10 min, and discard the supernatant. Add 200 μL of 1% Triton X‐100, and lyse for 40 min. The lysed liquid is measured directly without centrifugation. Use a reagent kit (Nanjing Jiangcheng Bioengineering Institute, China) to detect the concentration of intracellular CHO and TG.

### 2.11 Oil red O staining

Discard the old culture medium, slowly add PBS to the edge of the well plate, and wash it twice. Fix with 4% paraformaldehyde at room temperature for 30 min. Wash the plate twice with PBS, and add a small amount of 60% isopropanol to cover the cells in the well plate for 20 s, discard the 60% isopropanol, and let the water dry slightly. Add Oil Red O working solution to the well plate to cover the cells, stain at room temperature in the dark for 30 min, and remove the staining solution. Add 60% isopropanol for rapid differentiation for 3 s, and wash it with pure water 3 times for 5 min each time. Add PBS to cover the cells, and observe them under a microscope.

### 2.12 BODIPY staining

Cells were seeded onto a 6‐well plate and stained with a cell density of 60%–80%. Wash twice with PBS, add 4% paraformaldehyde and fix at room temperature for 15 min. Wash the cells twice with PBS, add an appropriate volume (fully covering the cells) of staining solution, and incubate at room temperature in the dark for 30 min. Aspirate the staining solution and wash the cells twice with PBS, add PBS to soak the cells, observe the fluorescence using a fluorescence microscope, and take photographs.

### 2.13 Statistical analysis

All data were processed and analyzed using Excel and GraphPad Prism 9.0 software. All experimental data were obtained from three independent biological replicates, and results are presented as mean ± SD. Comparisons between two groups were performed using an independent samples *t*‐test, whereas comparisons among multiple groups were analyzed using one‐way or two‐way ANOVA. Statistical significance is indicated by asterisks as follows: ns: not statistically significant, *p < 0.05, **p < 0.01, ***p < 0.001, and ****p < 0.0001.

## 3 RESULTS

### 3.1 Histone Kla is associated with sorafenib resistance in HCC

**FIGURE 1:**
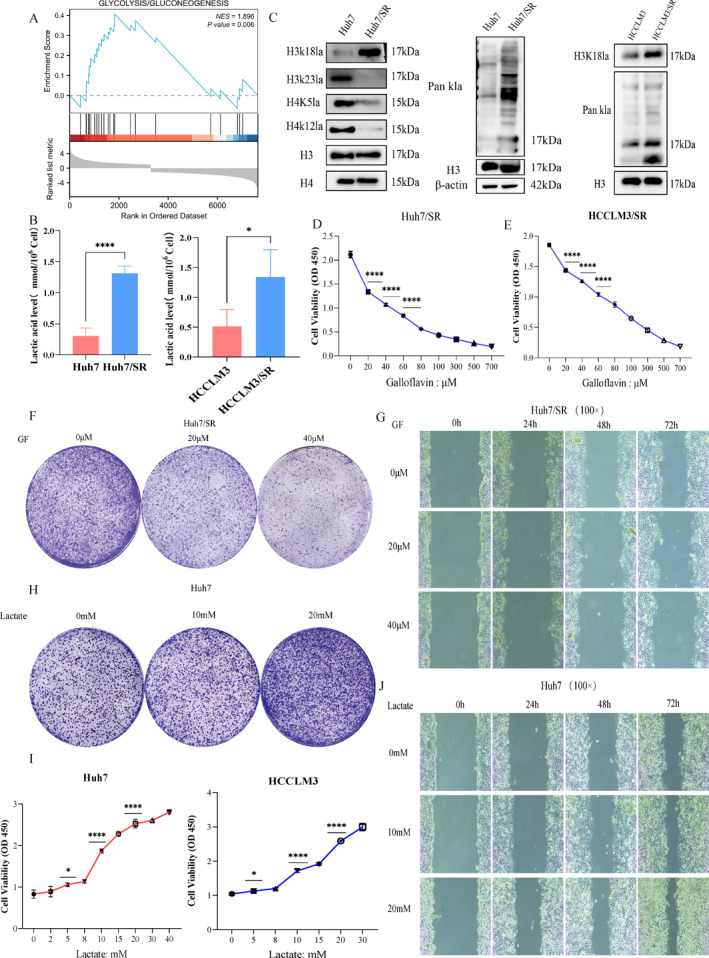
Histone Kla influences sorafenib resistance in HCC. (A) Gene set enrichment analysis. (B) Lactic acid content measurement. (C) Kla level detection. (D–G) Effects of GF treatment on cell proliferation and migration. (H–J) Effects of sodium lactate treatment on cell proliferation and migration. *n* = 3 (independent biological replicates and representative bands shown by western blot).*p < 0.05,****p < 0.0001.

A growing body of evidence suggests that elevated lactate levels can influence sensitivity to anticancer drugs via histone Kla. To investigate the role of histone Kla in sorafenib resistance in HCC, we downloaded the sorafenib resistance dataset (GSE213615) from Gene Expression Omnibus (GEO) and performed differential expression analysis. Gene set enrichment analysis (GSEA) was conducted on the differentially expressed genes (|log2FC| > 1.5 and *p <* 0.05), revealing significant enrichment of glycolysis‐related metabolic pathways (Figure 1A). Subsequently, lactate levels in sorafenib‐sensitive cells (Huh7 and HCCLM3) and sorafenib‐resistant cells (Huh7/SR and HCCLM3/SR, IR > 2) were measured using a lactate assay kit. Results showed a significant increase in lactate content in Huh7/SR and HCCLM3/SR cells (Figure 1B). Western blotting further demonstrated significantly elevated levels of H3K18la and pan‐lysine lactylation (pan‐Kla) in resistant cells (Figure 1C). To establish a causal relationship between histone Kla and sorafenib resistance in HCC, resistant cells were treated with the glycolysis inhibitor 2‐deoxy‐D‐glucose (2‐DG) and the lactate dehydrogenase (LDH) inhibitor galloflavin (GF). Both treatments significantly inhibited the proliferation and migration capacities of resistant cells (Figure 1D–G and Figure S1A–H). Additionally, treatment of sensitive cells with sodium lactate (Lac) promoted cell proliferation and migration (Figure 1H–J and Figure S1I and J). Collectively, these data indicate that histone Kla significantly contributes to the development of sorafenib resistance in HCC.

### 3.2 Histone Kla mediates lipid metabolism reprogramming and affects sorafenib resistance in HCC

**FIGURE 2:**
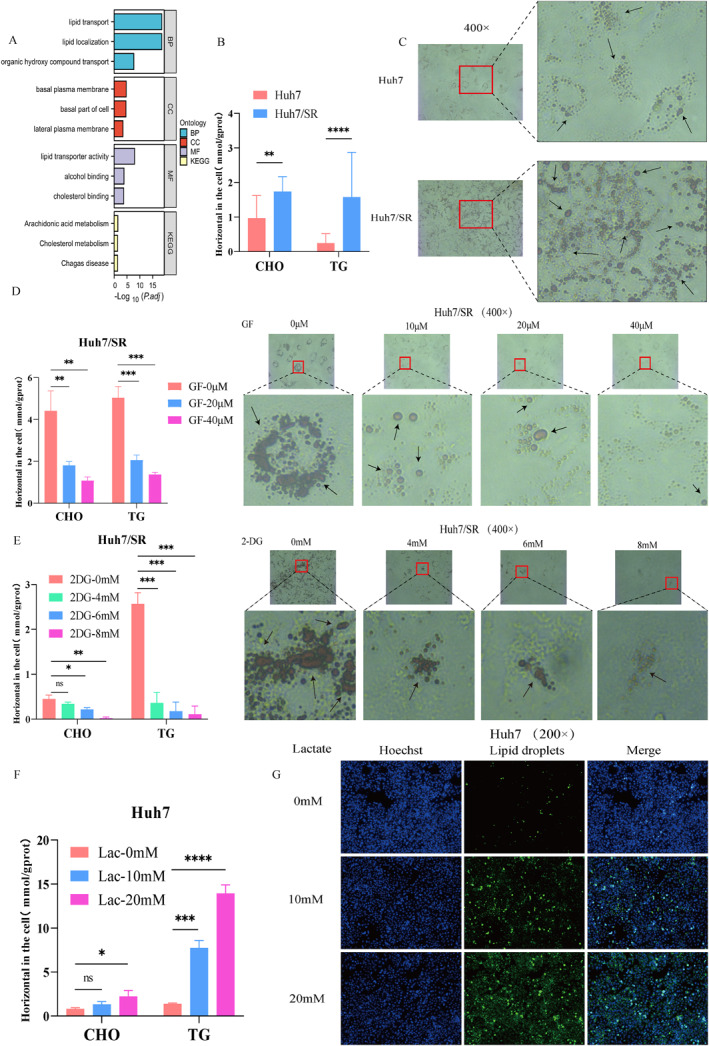
Histone Kla drives lipid metabolism reprogramming. (A) GO‐KEGG analysis of sorafenib resistance–associated genes in HCC. (B and C) Differences in CHO, TG, and lipid droplet between sensitive and resistant cells. (D and E) Effects of GF or 2‐DG treatment on lipid metabolism. (F and G) Effects of sodium lactate treatment on lipid metabolism. *n* = 3 (independent biological replicates; results expressed as mean ± SD). ns: not statistically significant, *p < 0.05, **p < 0.01, ***p < 0.001, and ****p < 0.0001.

Previous studies have demonstrated a close association between glycolysis, dysregulated lipid metabolism, and the development of tumor drug resistance.^9^, ^19^ Therefore, we performed Gene Ontology (GO) and Kyoto Encyclopedia of Genes and Genomes (KEGG) enrichment analyses on differentially expressed molecules associated with sorafenib resistance in HCC (|log2FC| > 1.5, *p <* 0.05). The results indicated that these molecules were significantly enriched in lipid metabolic processes (Figure 2A). Next, we assessed lipid metabolism levels in sensitive and resistant cells. Compared with sensitive cells, resistant cells exhibited significantly increased levels of CHO and TG (Figure 2B, Figure S2A), along with a marked increase in LD accumulation (Figure 2C, Figure S2B).

**FIGURE 3:**
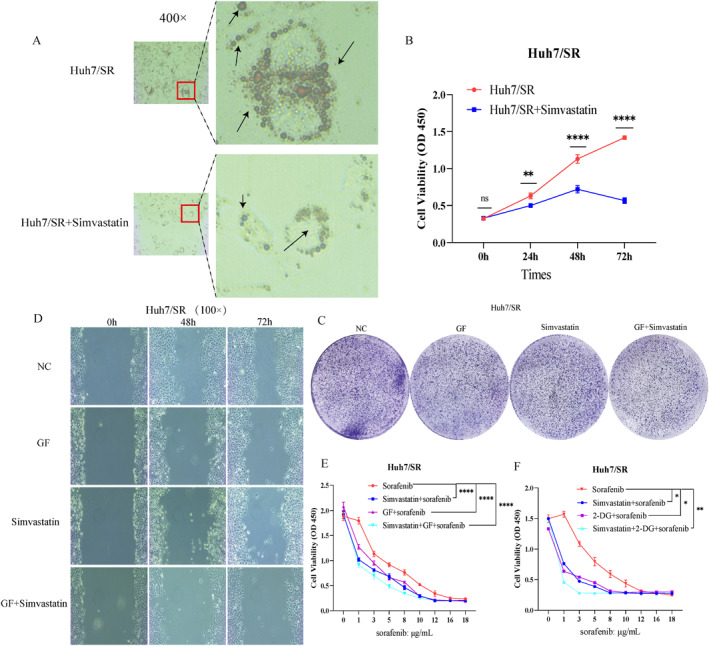
Histone Kla‐driven lipid metabolism reprogramming affects sorafenib resistance in HCC. (A) Effect of simvastatin treatment on lipid droplet accumulation. (B) Effect of simvastatin treatment on cell proliferation. (C and D) Effects of simvastatin combined with GF on cell proliferation and migration. (E and F) Changes in cell sensitivity to sorafenib after co‐administration of simvastatin with GF or 2‐DG. *n* = 3 (independent biological replicates). ns: no statistical significance; *p < 0.05, **p < 0.01, and ****p < 0.0001.

Based on these findings, we investigated whether histone Kla could act as an upstream regulator driving lipid metabolism reprogramming. Resistant cells were treated with 2‐DG or GF, whereas sensitive cells were treated with sodium lactate. The results showed that CHO, TG, and LD levels in resistant cells were significantly reduced following 2‐DG or GF treatment (Figure 2D and 2E and Figure S2C and D), whereas sodium lactate treatment produced the opposite effect (Figure 2F and 2G and Figure S2E and 2F). Next, we examined the impact of histone Kla‐mediated lipid metabolism reprogramming on the biological functions of sorafenib‐resistant HCC cells. Cells were treated with the lipid synthesis inhibitor simvastatin, which significantly reduced LD accumulation and suppressed the proliferative capacity of resistant cells (Figure 3A and 3B and Figure S3A and B). Furthermore, combined treatment with simvastatin and either 2‐DG or GF further inhibited cell proliferation and migration (Figure 3C and 3D and Figure S3C and D) and enhanced sensitivity to sorafenib (Figure 3E and 3F and Figure S3E and F). These findings indicate that histone Kla promotes lipid metabolism reprogramming and lipid accumulation, thereby contributing to sorafenib resistance in HCC.

### 3.3 HOXB13 is a key effector molecule in histone Kla–driven lipid metabolism reprogramming

**FIGURE 4:**
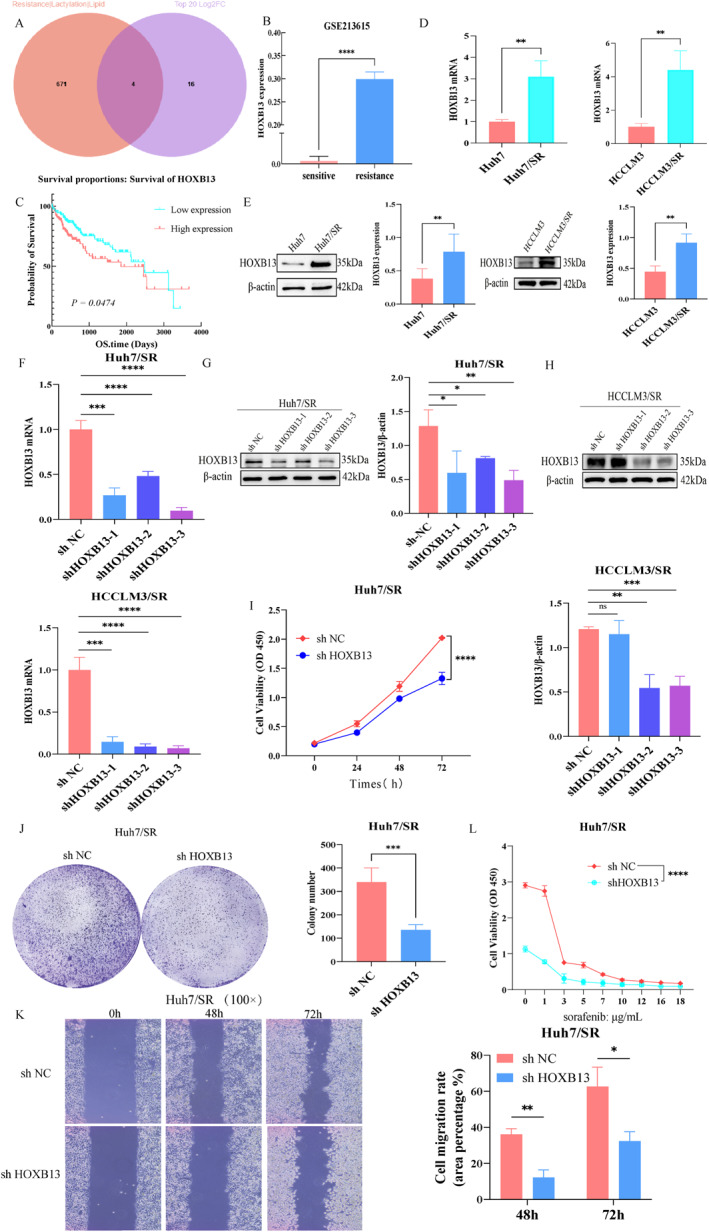
HOXB13 is a key effector molecule in histone Kla‐driven lipid metabolism reprogramming. (A) Venn analysis of the Top 20 Log2FC genes and the 675 sorafenib resistance–related molecules in HCC. (B) Differential expression of GSE213615 HOXB13 mRNA. (C) Prognostic analysis of HOXB13 in the TCGA_LIHC dataset (*p =* 0.0474). (D, E) Differential analysis of HOXB13 expression in sensitive and resistant cells. (F–H) HOXB13 knockdown validation. (I–L) The effect of shHOXB13 on cellular biological activity. *n* = 3 (independent biological replicates, results expressed as mean ± SD; representative bands shown by western blot). ns: no statistical significance; *p < 0.05, **p < 0.01, ***p < 0.001, and ****p < 0.0001.

To explore the molecular mechanisms underlying histone Kla–mediated lipid metabolism reprogramming, we downloaded H3K18la modification ChIP‐seq datasets (GSE207814 and GSE242013), lipid metabolism–related datasets (GSE248903 and GSE204898), and relevant molecules from the literature (PMC9455632 and PMC9873467) from the GEO database. The data were processed using R, and Venn analysis was performed to integrate H3K18la target genes, lipid metabolism–related genes, and sorafenib resistance–associated genes in HCC (|log2FC| > 1.5, *p <* 0.05), yielding 675 candidate molecules (Figure S4A). To further refine the candidate list, a second Venn analysis was conducted between these 675 molecules and the top 20 differentially expressed genes associated with sorafenib resistance (Log2FC and *p <* 0.05), identifying four candidate genes (Figure 4A, Table S3). Among them, ALOX15B and HOXB13 are both upregulated in drug‐resistant datasets and the TCGA_LIHC tumor cohort (Figure 4B and Table S3). Previous studies have found that HOXB13 can serve as a potential biomarker for poor prognosis in HCC, but its role in sorafenib resistance in HCC is still unclear. Moreover, further analysis also indicates that high HOXB13 expression was associated with poor prognosis in patients with liver cancer (Figure 4C). Therefore, we selected it as a candidate molecule for subsequent mechanistic studies.

We next investigated the functional role of HOXB13 in sorafenib resistance in HCC. RT‐qPCR and western blot analyses demonstrated that HOXB13 was significantly upregulated in resistant cells (Figure 4D–E). Subsequently, three shHOXB13 sequences were constructed and transfected into cells, with Sequence 3 showing the highest knockdown efficiency (Figure 4F–H). Functional assays, including CCK‐8, colony formation, and wound healing assays, showed that HOXB13 knockdown significantly inhibited the proliferation and migration of resistant cells (Figure 4I–K and Figure S4B–D) and markedly increased their sensitivity to sorafenib (Figure 4L and Figure S4E). These results suggest that HOXB13 plays a critical role in the development of sorafenib resistance in HCC, potentially regulated by H3K18la modification.

### 3.4 HOXB13 is a key downstream target mediating H3K18la‐driven sorafenib resistance in HCC

**FIGURE 5:**
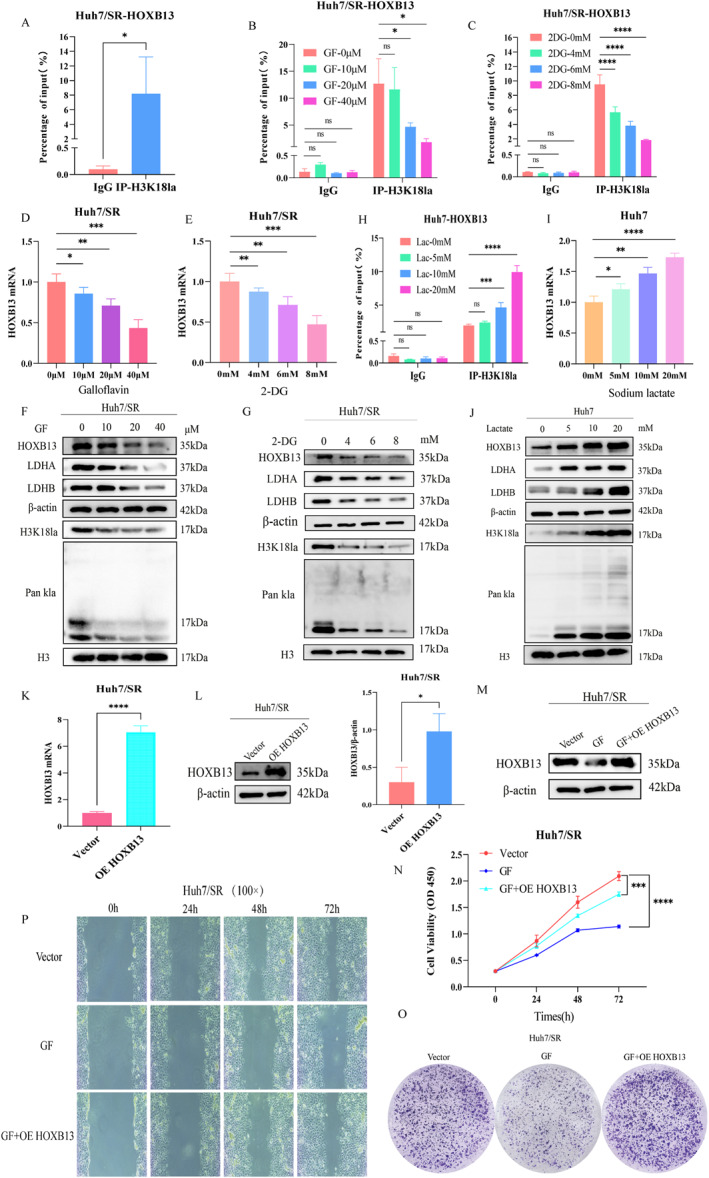
Histone Kla regulates the transcriptional activation of HOXB13. (A) Enrichment analysis of H3K18la modification in the HOXB13 promoter region. (B and C) Effects of 2‐DG or GF treatment on H3K18la modification enrichment. (D–G) Effects of 2‐DG or GF treatment on HOXB13 expression. (H) Effect of sodium lactate treatment on H3K18la modification enrichment. (I and J) Effects of sodium lactate treatment on HOXB13 expression. (K and L) Validation of HOXB13 overexpression efficiency. (M–P) Histone Kla regulates HOXB13 expression and participates in sorafenib resistance in HCC. *n* = 3 (independent biological replicates, results expressed as mean ± SD; representative bands shown by western blot). ns: no statistical significance; *p < 0.05, **p < 0.01, ***p < 0.001, ****p < 0.0001.

Based on these findings, we investigated the molecular mechanisms by which H3K18la modifies HOXB13. MNase ChIP‐qPCR experiments revealed significant enrichment of H3K18la between 1 and 2 kb upstream of the HOXB13 transcription start site (Figure 5A), suggesting a direct role in transcriptional activation. Further analyses demonstrated a positive correlation between H3K18la enrichment and HOXB13 expression. Treatment with 2‐DG or GF reduced H3K18la enrichment in the HOXB13 promoter region and significantly decreased HOXB13 mRNA and protein expression (Figure 5B–G and Figure S5 A–D), whereas treatment with sodium lactate produced the opposite effects (Figure 5H–J, Figure S5E,F).

To verify that HOXB13 is a critical downstream effector in H3K18la‐mediated sorafenib resistance, we constructed and transfected cells with an HOXB13 overexpression (OE HOXB13) plasmid. RT‐qPCR and western blot results confirmed significant HOXB13 upregulation following transfection (Figure 5K–L). Subsequently, a rescue experiment demonstrated that OE HOXB13 effectively reversed the inhibitory effects of GF treatment on the biological functions of sorafenib‐resistant cells (Figure 5M–P). These results indicate that H3K18la activates HOXB13 transcription by modulating chromatin structure, thereby contributing to sorafenib resistance in HCC.

### 3.5 Lipometabolism reprogramming is critical for HOXB13‐mediated sorafenib resistance in HCC

**FIGURE 6:**
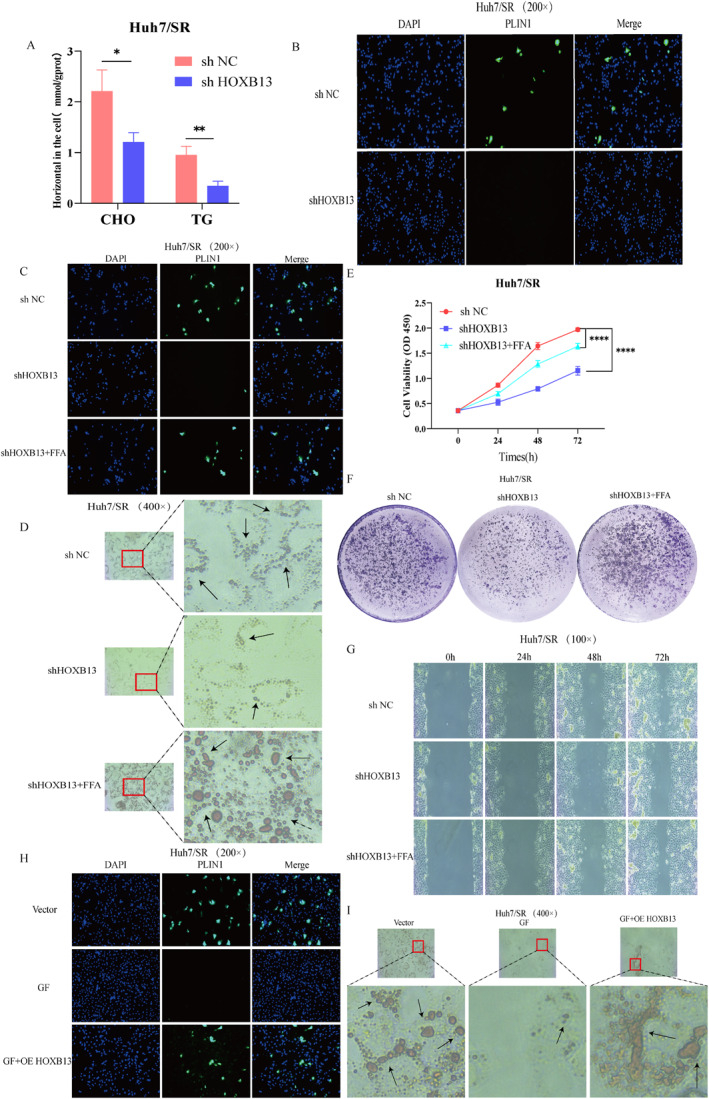
Lipid metabolism reprogramming is a key step in HOXB13‐mediated drug resistance. (A and B) Effects of shHOXB13 on cellular lipid metabolism. (C and D) Effects of shHOXB13 combined with free fatty acids treatment on PLIN1 expression and lipid droplet. (E–G) Effects of shHOXB13 combined with FFA treatment on cell proliferation and migration. (H and I) Effects of OE HOXB13 on PLIN1 expression and LD levels after GF treatment. *n* = 3 (independent biological replicates, results expressed as mean ± SD). ns: no statistical significance;*p < 0.05, **p < 0.01,and ****p < 0.0001. [Correction added on 17 May 2026, after first online publication: The Figure 6 has been updated in this version.]

We next investigated the regulatory role of HOXB13 in lipid metabolism reprogramming. Drug‐resistant cells were transfected with shHOXB13, and changes in lipid metabolism‐related phenotypes were examined. Results showed that CHO and TG levels decreased significantly in shHOXB13‐treated cells (Figure 6A and Figure S6A), and perilipin 1 (PLIN1) expression was markedly reduced (Figure 6B). Furthermore, Oil Red O staining revealed significantly decreased LD accumulation following shHOXB13 transfection (Figure S6B and C). These findings indicate that HOXB13 promotes lipid accumulation by driving lipid metabolism reprogramming.

A rescue experiment was subsequently performed to investigate the mechanisms through which HOXB13‐driven lipid metabolism reprogramming affects sorafenib resistance. shHOXB13‐treated cells were incubated with 0.5 mmol/L free fatty acids (FFA), which restored PLIN1 expression and LD numbers (Figure 6C and 6D) and reversed the suppression of proliferation and migration induced by HOXB13 knockdown (Figure 6E–G). Furthermore, we examined whether histone Kla regulates lipid metabolism via HOXB13. Drug‐resistant cells treated with GF underwent HOXB13 overexpression, which reversed GF‐mediated inhibition of LD formation and PLIN1 expression, as demonstrated by Oil Red O and BODIPY staining and immunofluorescence (IF) assays (Figure 6H and 6I and Figure S6D). Collectively, these results indicate that H3K18la promotes sorafenib resistance in HCC by driving lipid metabolism reprogramming through HOXB13 regulation.

### 3.6 Activation of HIF‐1 signaling pathway promotes sorafenib resistance in HCC

**FIGURE 7:**
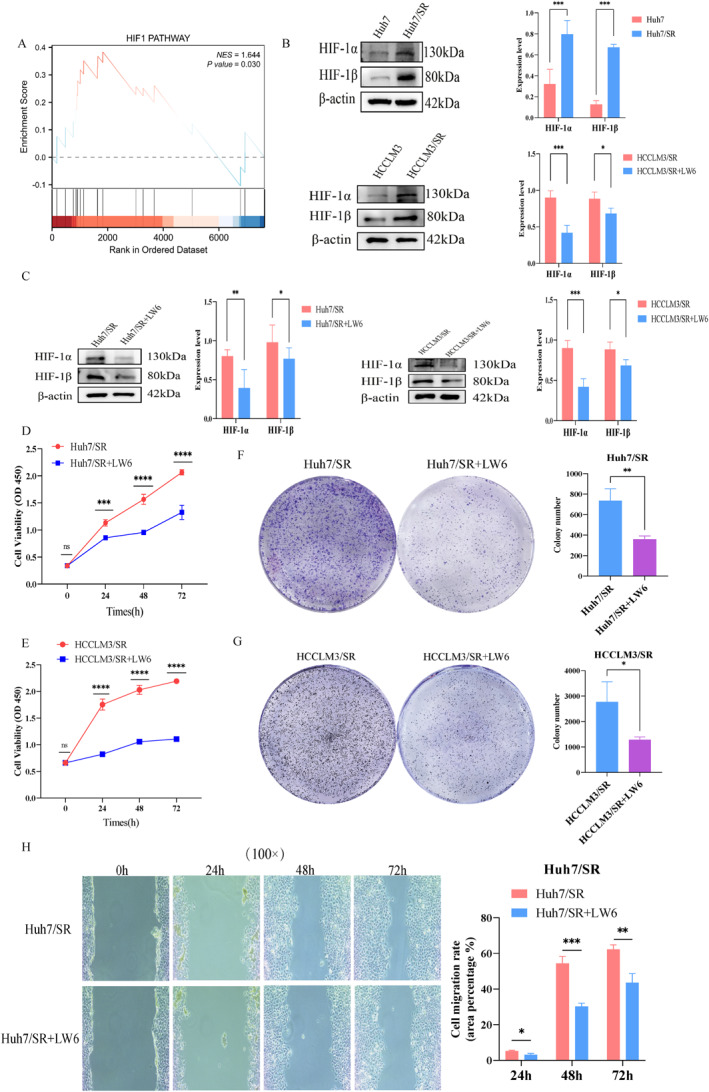
Activation of the HIF‐1 signaling pathway promotes sorafenib resistance in HCC. (A) Gene set enrichment analysis. (B) Analysis of HIF‐1 signaling pathway activation in sensitive and drug‐resistant cells. (C) Effects of LW6 treatment on the expression of HIF‐1 signaling pathway–related molecules. (D–H) Impact of HIF‐1 signaling pathway inhibition on cellular biological functions. *n* = 3 (independent biological replicates, results expressed as mean ± SD; representative bands shown by western blot). ns: no statistical significance; *p < 0.05, **p < 0.01, ***p < 0.001, and ****p < 0.0001.

Previous studies have shown that activation of the HIF‐1 signaling pathway is associated with lactate and lipid metabolic reprogramming as well as tumor resistance.^20^, ^21^, ^22^, ^23^, ^24^ Therefore, we hypothesize that HIF‐1 may play a role in sorafenib resistance in HCC. Based on this, we conducted GSEA analysis on the differentially expressed genes related to sorafenib resistance in HCC and found that the HIF‐1 signaling pathway could be significantly enriched (Figure 7A). Western blot analysis further revealed significantly increased levels of HIF‐1α and HIF‐1β, molecules related to the HIF‐1 signaling pathway, in resistant cells (Figure 7B). The above results indicate that the HIF‐1 signaling pathway is significantly activated in resistant cells. Next, we examined the effects of HIF‐1 signaling pathway inhibition on sorafenib resistance in HCC. Treatment with the HIF‐1 signaling pathway inhibitor LW6 significantly reduced the protein expression of HIF‐1α and HIF‐1β in resistant cells (Figure 7C), indicating suppression of pathway activity. Cell viability assays showed reduced proliferation of resistant cells following LW6 treatment (Figure 7D and 7E). Colony formation assays revealed a significant reduction in long‐term proliferative capacity upon HIF‐1 pathway inhibition (Figure 7F and 7G). Moreover, wound healing assays demonstrated impaired cell migration after HIF‐1 pathway inhibition (Figure 7H). These findings indicate that activation of the HIF‐1 signaling pathway contributes to sorafenib resistance in HCC.

### 3.7 The interaction between HOXB13 and HIF‐1 α affects activation of the HIF‐1 signaling pathway

**FIGURE 8:**
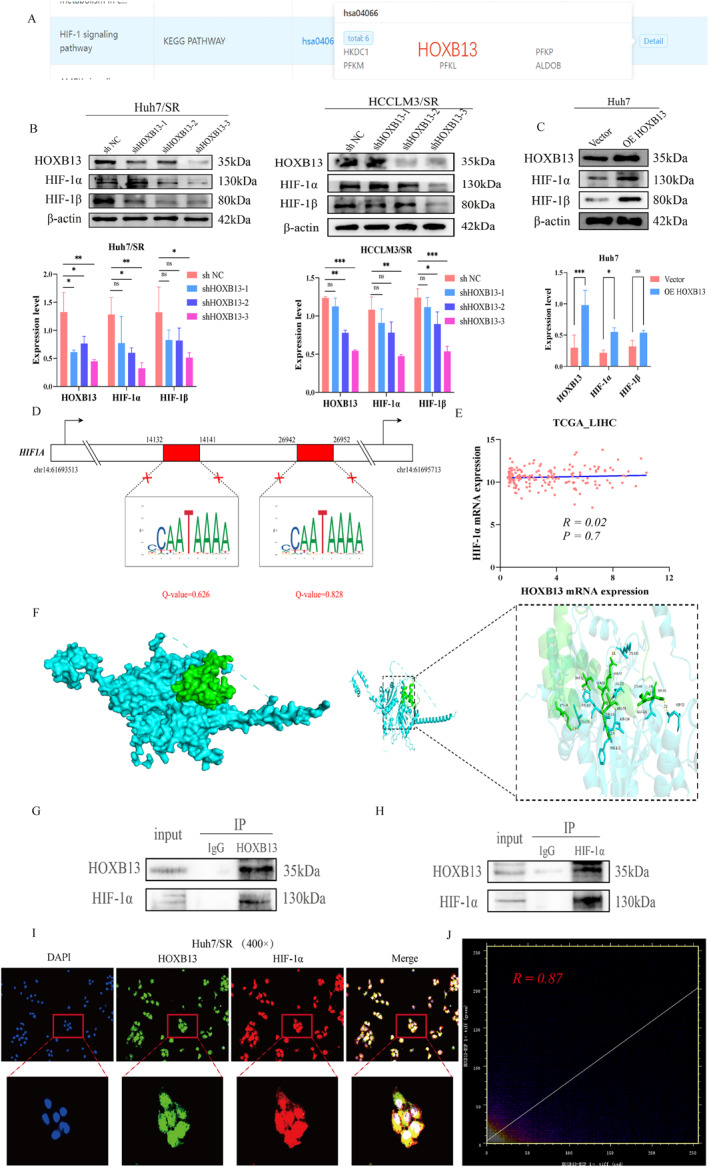
Interaction between HOXB13 and HIF‐1α affects HIF‐1 signaling pathway activation. (A) Kyoto Encyclopedia of Genes and Genomes pathway enrichment analysis. (B, C) Effects of HOXB13 on HIF‐1 signaling pathway activation. (D) Prediction of HOXB13 binding to the HIF‐1α promoter region. (E) Co‐expression analysis of HOXB13 and HIF‐1α mRNA. (F) Molecular docking of HOXB13 with HIF‐1α. (G and H) Binding analysis of HOXB13 to HIF‐1α. (I) Colocalization analysis of HOXB13 and HIF‐1α. (J) Pearson correlation analysis of HOXB13 and HIF‐1α fluorescence colocalization. *n* = 3 (independent biological replicates, results expressed as mean ± SD; representative experimental results shown in Co‐IP and IF colocalization experiments). ns: no statistical significance; *p < 0.05, **p < 0.01, and ***p < 0.001.

As a key effector molecule that drives lipid metabolic reprogramming via histone Kla, HOXB13 may participate in activating the HIF‐1 signaling pathway. KEGG pathway enrichment analysis of molecules associated with sorafenib resistance in HCC revealed that HOXB13 was significantly enriched in the HIF‐1 signaling pathway (Figure 8A). Next, we investigated the regulatory relationship between HOXB13 and the HIF‐1 signaling pathway. After HOXB13 knockdown in drug‐resistant cells, the protein expression of HIF‐1 pathway–related molecules, including HIF‐1α and HIF‐1β, was markedly decreased under normoxic conditions (Figure 8B). Conversely, OE HOXB13 significantly increased HIF‐1α and HIF‐1β protein levels in sensitive cells (Figure 8C). These results indicate that HOXB13 may be involved in activating the HIF‐1 signaling pathway.

Given that activation of the HIF‐1 signaling pathway largely depends on the stability and nuclear localization of the HIF‐1α protein, we explored the molecular mechanisms by which HOXB13 regulates HIF‐1α. We selected a region from 2000 bp upstream to 200 bp downstream of the HIF‐1α gene transcription start site and used the AnimalTFDB v4.0 database to predict potential HOXB13 binding sites in the HIF‐1α promoter region. Under the established significance threshold (*Q‐value* < 0.05), no significant HOXB13 binding sites were detected in the target region (*Q‐value* > 0.05, Figure 8D, and Table S4), suggesting that HOXB13 may not directly regulate HIF‐1α transcription. To further confirm this inference, correlation analysis of HOXB13 and HIF‐1α mRNA expression levels was conducted using TCGA_LIHC data, revealing no significant correlation in HCC tissues (*R = 0*.*02* and *p >* 0.05, Figure 8E). Thus, in sorafenib‐resistant HCC cells, HOXB13 may regulate HIF‐1α through indirect mechanisms involving protein–protein interactions rather than classical transcriptional activation.

Based on these findings, we utilized the HDOCK online tool for molecular docking analysis between HOXB13 and HIF‐1α proteins. The results revealed a potential interaction (docking score = −258.05 and confidence score = 0.8967, Figure 8F), indicating possible complex formation through protein–protein interactions. We subsequently validated this interaction using bidirectional Co‐IP experiments. Western blot analysis clearly demonstrated the presence of HIF‐1α protein in complexes immunoprecipitated with an HOXB13‐specific antibody (Figure 8G). Conversely, HOXB13 protein was also effectively co‐precipitated using an HIF‐1α‐specific antibody (Figure 8H). These results confirm a specific intracellular protein–protein interaction between HOXB13 and HIF‐1α. To further elucidate the subcellular localization of this interaction, IF localization experiments were performed. The results showed significant colocalization of HOXB13 and HIF‐1α predominantly within the nucleus (*R =* 0.87), although some colocalization was also observed in the cytoplasm (Figure 8I and 8J, Table S5). This suggests that their interaction mainly occurs in the nucleus but may also involve dynamic interactions and transport processes within the cytoplasm. Collectively, these findings demonstrate that HOXB13 activates the HIF‐1 signaling pathway through direct protein–protein interactions with HIF‐1α, promoting nuclear accumulation, and thereby contributing to the development of sorafenib resistance in HCC.

### 3.8 H3K18la/HOXB13/HIF‐1 axis regulates lipid metabolism reprogramming in sorafenib‐resistant HCC cells

**FIGURE 9:**
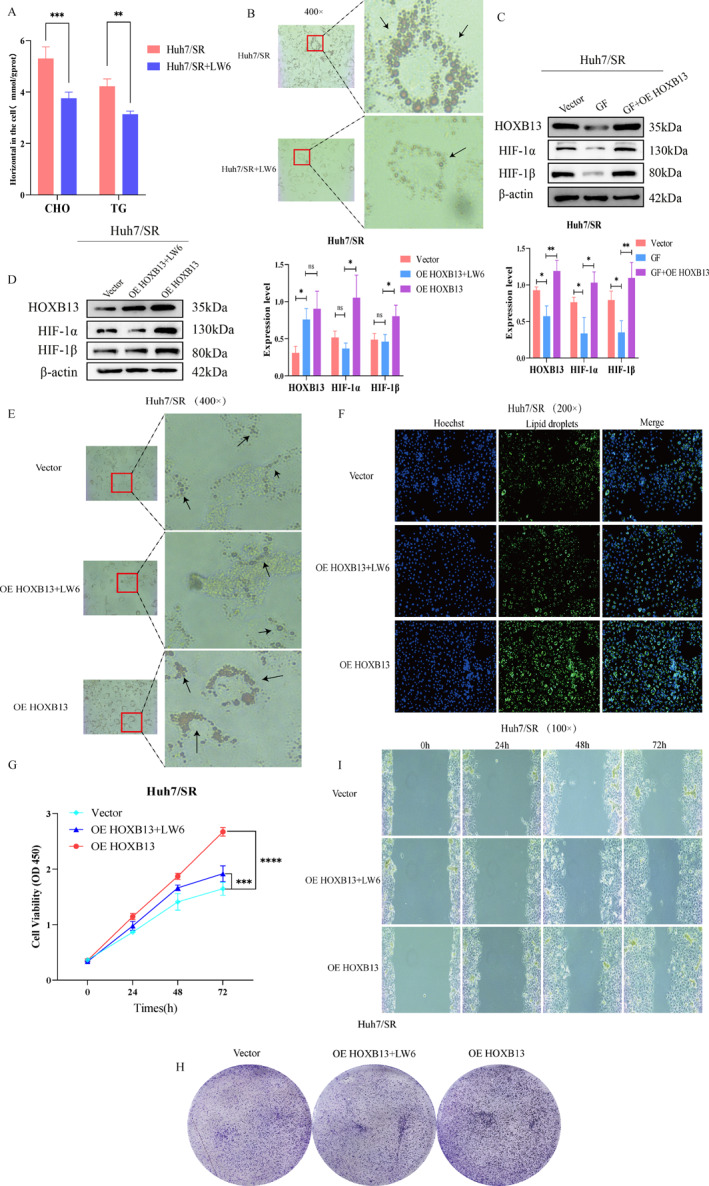
H3K18la/HOXB13/HIF‐1 axis reprogramming lipid metabolism in HCC drug‐resistant cells. (A and B) Effects of HIF‐1 signaling pathway inhibition on lipid metabolism. (C) Effects of OE HOXB13 on the HIF‐1 signaling pathway after GF treament. (D) Effects of LW6 treatment on the HIF‐1 signaling pathway after OE HOXB13. (E–I) Effects of LW6 treatment after OE HOXB13 on cellular lipid metabolism and biological functions. *n* = 3 (independent biological replicates, results expressed as mean ± SD). ns: no statistical significance; *p < 0.05, **p < 0.01, ***p < 0.001, and ****p < 0.0001.

Finally, we investigated the impact of lipid metabolism reprogramming driven by the H3K18la/HOXB13/HIF‐1 axis on sorafenib resistance in HCC. We treated resistant cells with LW6, an inhibitor of the HIF‐1 signaling pathway, and observed that LW6 significantly reduced CHO, TG, and LD levels in these cells (Figure 9A,B and Figure S7A and B). In other words, inhibition of the HIF‐1 signaling pathway markedly suppressed lipid accumulation.

Next, a rescue experiment was designed to clarify how H3K18la promotes sorafenib resistance in HCC through lipid metabolism reprogramming mediated by the HOXB13/HIF‐1 axis. First, HOXB13 was overexpressed in cells exhibiting suppression of H3K18la. Western blot results demonstrated that HOXB13 overexpression restored protein expression levels of HIF‐1α and HIF‐1β, key molecules associated with the HIF‐1 signaling pathway (Figure 9C). This indicated that H3K18la modulates activation of the HIF‐1 signaling pathway by regulating HOXB13 expression. Subsequently, LW6 treatment was applied to OE HOXB13 cells, revealing that LW6 inhibited HOXB13‐induced activation of the HIF‐1 signaling pathway (Figure 9D). Oil Red O and BODIPY staining results confirmed that LW6 partially reversed lipid accumulation induced by HOXB13 overexpression (Figure 9E and 9F, Figure S7C). Furthermore, functional cellular assays demonstrated that LW6 treatment effectively reversed HOXB13‐induced HIF‐1 pathway activation, thereby rescuing proliferation, migration, and other drug‐resistant phenotypes promoted by HOXB13 overexpression (Figure 9G–I). Collectively, these data indicate that H3K18la drives lipid metabolism reprogramming through the HOXB13/HIF‐1 signaling axis, resulting in lipid accumulation and promoting sorafenib resistance in HCC.

## 4 DISCUSSION

Lactic acid, a key product of the Warburg effect, accumulates at high levels in metabolically active tumor cells and plays an important role in promoting tumor progression.^31^, ^32^ Recent studies indicate that lactate can act as a substrate for posttranslational protein modifications, thereby contributing to cancer development and progression and being associated with chemotherapy resistance^33^, ^34^, ^35^ Therefore, we measured lactate levels in sorafenib‐resistant and sensitive HCC cells. The results showed that lactate levels were significantly elevated in sorafenib‐resistant cells compared with sensitive cells, indicating enhanced glycolytic activity in resistant cells. Based on these findings, our study further identifies a novel lactate‐driven, epigenetically mediated mechanism of sorafenib resistance, in which lactate promotes H3K18la modification, leading to upregulation of HOXB13 expression and activation of the HIF‐1 signaling pathway, thereby driving lipid metabolic reprogramming and ultimately conferring sorafenib resistance in HCC cells.

Histone PTMs serve as a central hub linking cellular metabolic status to gene transcriptional regulation. Among these, classical PTMs such as acetylation (Kac) and methylation (Kme) have been shown to influence tumor drug resistance by modulating chromatin accessibility.^36^, ^37^, ^38^, ^39^, ^40^ Histone Kla, a recently identified PTM first reported in 2019, directly utilizes lactate as a substrate to mediate epigenetic regulation, thereby establishing a direct connection between cellular metabolism and gene transcription,^7^ and contributing to tumor drug resistance. Therefore, we examined Kla levels in HCC cells resistant to and sensitive to sorafenib and found that H3K18la and pan‐Kla levels were significantly elevated in resistant cells. Next, we investigated whether histone Kla contributes to the development of sorafenib resistance in HCC. Resistant cells were treated with the glycolysis inhibitor 2‐DG. Given that 2‐DG, as a nonspecific glycolysis inhibitor, may exert effects through lactate‐independent mechanisms, we additionally employed the LDH‐specific inhibitor GF to validate the role of lactate inhibition. The results showed that both treatments reduced H3K18la and pan‐Kla levels and suppressed the proliferation and migration of resistant cells. In contrast, treatment of sorafenib‐sensitive HCC cells with sodium lactate produced opposite effects. These findings indicate that histone Kla plays a critical role in the development of sorafenib resistance in HCC, and that targeting histone Kla may enhance the sensitivity of HCC cells to sorafenib. Therefore, further investigation of the specific mechanisms by which histone Kla regulates sorafenib resistance in HCC is warranted.

Histone Kla modification, a central epigenetic mechanism linking the Warburg effect to tumor drug resistance, mediates tumor resistance by regulating gene transcriptional activation and participates in multiple pathways, including DNA repair, ferroptosis, tumor cell stemness, and the immune microenvironment.^41^, ^42^, ^43^, ^44^, ^45^ Yue et al.^46^ demonstrated that H3K9la modification significantly enriches the LUC7L2 promoter region, activating its expression, inducing retention of intron 7 in the MLH1 gene, downregulating MLH1 protein levels, impairing mismatch repair function, and consequently promoting resistance to temozolomide. These findings suggest that histone Kla‐mediated regulation of gene transcription may be a general mechanism underlying tumor drug resistance. Therefore, we explored the molecular mechanisms by which histone Kla affects sorafenib resistance in HCC. We performed Venn analysis of genes associated with sorafenib resistance in HCC and genes modified by histone Kla, combined with differential and prognostic analyses, to identify potential key molecules. Our results indicated that HOXB13, as an H3K18la‐modified target gene, was significantly upregulated in the sorafenib‐resistant HCC dataset, and its high expression correlated with poor prognosis in liver cancer patients. Thus, we selected HOXB13 as the core target for further mechanistic studies investigating its role and regulatory mechanisms in histone Kla‐mediated sorafenib resistance in HCC. Our data showed that HOXB13 expression was significantly elevated in sorafenib‐resistant HCC cells. Treatment with 2‐DG or GF decreased both mRNA and protein levels of HOXB13, while sodium lactate produced the opposite effect, suggesting histone Kla may regulate HOXB13 expression. Subsequently, MNase ChIP‐qPCR assays confirmed enrichment of H3K18la within the HOXB13 promoter region, facilitating its transcriptional activation and increased expression. Collectively, these findings establish a critical molecular connection between histone Kla and targeted therapy resistance in HCC, providing a novel molecular target for clarifying the specific mechanisms underlying sorafenib resistance.

HOXB13 belongs to the HOX family of transcription factors, which previous studies have implicated in the regulation of tumor cell proliferation, invasion, migration, and drug resistance through interactions with target genes.^47^, ^48^, ^49^, ^50^ For example, HOXB13 inhibits tamoxifen mediated transcriptional inhibition through interaction with estrogen receptor α (ER α), and finally endows breast cancer cells with tamoxifen resistance.^51^ Tang et al.^52^ demonstrated that hyperactivation of the HOXB13/PIMREG axis promotes HCC progression and resistance to 5‐FU. In this study, we found that HOXB13 knockdown reduced proliferation and migration in drug‐resistant cells, while HOXB13 overexpression in cells previously inhibited by histone Kla reversed the suppressive effects of GF treatment on drug‐resistant phenotypes. These findings suggest that HOXB13 plays a central role in connecting histone Kla modification with sorafenib resistance in HCC. Therefore, we further explored the downstream mechanisms by which HOXB13 influences sorafenib resistance. Through GSEA and KEGG pathway enrichment analyses, we identified significant enrichment of HOXB13 within the HIF‐1 signaling pathway. The HIF‐1 pathway serves as a central transcriptional mediator of tumor adaptation to hypoxic and metabolic stress, promoting angiogenesis, glycolysis, and therapeutic resistance by regulating target genes such as VEGF and GLUT1.^22^, ^23^, ^24^ Although classical studies have mainly focused on the established mechanism of hypoxia‐induced HIF‐1α protein stabilization,^53^, ^54^, ^55^ recent evidence suggests that epigenetic modifications, including H3K27ac and H3K4me3, can activate the HIF‐1 pathway by regulating HIF‐1α transcription or recruiting co‐activators under normoxic conditions.^56^, ^57^ Our study expands this understanding by demonstrating that the HIF‐1 signaling pathway is significantly activated in sorafenib‐resistant HCC cells and that the expression levels of HIF‐1 pathway‐related molecules HIF‐1α and HIF‐1β positively correlate with HOXB13 expression. We next investigated the molecular mechanisms underlying HOXB13‐mediated regulation of the HIF‐1 pathway. Given that HIF‐1 pathway activation primarily depends on the stability and nuclear localization of HIF‐1α protein, we conducted bioinformatics analyses and found no significant direct binding or expression correlation between HOXB13 and the HIF‐1α promoter. This suggests that HOXB13 may regulate HIF‐1α indirectly through protein–protein interactions rather than classical transcriptional activation. Based on this, protein–protein docking and experimental validation demonstrated a specific direct interaction between HOXB13 and HIF‐1α proteins under normoxic conditions. IF colocalization assays further revealed that HOXB13 and HIF‐1α significantly colocalize mainly within the nucleus, with additional partial colocalization observed in the cytoplasm. These results suggest that their interaction primarily occurs in the nucleus, though dynamic binding and nuclear transport processes may also occur in the cytoplasm. Based on these findings and the classical model of HIF‐1α stabilization, we propose that HOXB13 may interact with HIF‐1α immediately following its synthesis, facilitating nuclear translocation and reducing VHL‐mediated ubiquitination and degradation in the cytoplasm.^58^ Furthermore, functional experiments confirmed that inhibition of the HIF‐1 signaling pathway significantly reversed HOXB13‐mediated resistance phenotypes, indicating that the H3K18la/HOXB13/HIF‐1 axis represents a central regulatory pathway in sorafenib resistance in HCC. This discovery expands the regulatory network controlling the HIF‐1 signaling pathway in tumors, revealing that epigenetically driven transcription factors, in addition to hypoxia, can serve as crucial activators of the HIF‐1 pathway.

Tumors are characterized not only by genetic but also by metabolic abnormalities. Metabolism reprogramming, particularly lipid metabolism reprogramming, has become a key feature of tumor drug resistance. Enhanced fatty acid synthesis and lipid accumulation confer chemotherapy and targeted drug resistance to tumor cells by supplying energy and protecting against cellular stress.^59^, ^60^, ^61^ Zhao et al.^62^ reported that HKDC1 reprograms lipid metabolism in gastric cancer cells by regulating fatty acid synthase mRNA stability through ribonucleoprotein complex formation, thereby promoting metastasis and cisplatin resistance. Our study demonstrates significantly higher levels of CHO, TG, and LD in sorafenib‐resistant HCC cells than sensitive cells. Treatment with simvastatin, a lipid synthesis inhibitor, effectively suppressed resistance phenotypes, indicating that aberrant lipid metabolism is a critical mechanism underlying sorafenib resistance in HCC. HIF‐1 is a recognized metabolic regulator; previous studies have demonstrated that HIF‐1α directly regulates the transcription of key enzymes involved in fatty acid synthesis and critical genes involved in cholesterol synthesis.^20^, ^21^ Additionally, HOXB13 has been reported to regulate metabolism‐related genes in multiple tumor types.^29^ Based on these findings, we focused on the central effector mechanism of the H3K18la/HOXB13/HIF‐1 axis in lipid metabolism reprogramming. Our experimental results showed that inhibition of the H3K18la/HOXB13/HIF‐1 axis significantly reduced the levels of CHO and TG, the number of LDs, and PLIN1 expression in drug‐resistant cells. Moreover, overexpression of HOXB13 or supplementation with exogenous FFA partially restored these markers, further confirming the pivotal role of lipid metabolism reprogramming downstream of this signaling axis. Integrating these findings with the established function of the HIF‐1 pathway, we propose that HIF‐1α interacts with HOXB13 in the cytoplasm, stabilizing HIF‐1α expression and facilitating its nuclear translocation. Subsequently, both transcription factors synergistically activate key genes involved in fatty acid and cholesterol synthesis, driving lipid metabolism reprogramming and ultimately conferring sorafenib resistance to HCC cells. This discovery closely integrates epigenetic regulation, stress signaling pathways, and lipid metabolism reprogramming, providing novel insights into the multidimensional molecular mechanisms underlying sorafenib resistance in HCC.

This study reveals a molecular mechanism mediated by H3K18la, which promotes sorafenib resistance in HCC through lipid metabolism reprogramming regulated by the HOXB13/HIF‐1 axis. H3K18la modification is enriched in the HOXB13 promoter region, activating its transcription. HOXB13 stabilizes HIF‐1α and activates the HIF‐1 signaling pathway through direct protein–protein interactions, thereby driving lipid metabolism reprogramming, lipid accumulation, and ultimately cellular resistance to sorafenib. However, this study has certain limitations. Firstly, we focused only on specific histone Kla modification sites. Notably, we observed significantly elevated Pan‐Kla levels in sorafenib‐resistant HCC cells; thus, the impact of nonhistone Kla modifications on sorafenib resistance cannot be ignored. Future studies should utilize Kla modification proteomics (Kla‐IP/MS) to identify key nonhistone Kla‐modified target proteins involved in drug resistance, establishing a comprehensive regulatory network encompassing both histone and nonhistone Kla modifications. Secondly, studies have shown that the H3K18la/HOXB13/HIF‐1 axis may serve as a biomarker for predicting sorafenib treatment response, but this hypothesis requires prospective validation in clinical patient cohorts. In addition, the HIF‐1 α inhibitor (LW6) and simvastatin used in this study showed inhibitory effects on sorafenib resistant HCC cells in an in vitro cell model, which may have the potential for combination therapy. However, this study is currently limited to the in vitro cellular level and lacks in vivo animal experiments for validation. Therefore, we plan to construct transplant tumor models or subcutaneous tumor models in subsequent studies to further evaluate the anti‐tumor efficacy and safety of HIF‐1 inhibitors or statins combined with sorafenib in vivo.

## 5 CONCLUSION

In summary, this study elucidates a critical mechanism by which H3K18la regulates sorafenib resistance in HCC. H3K18la transcriptionally activates HOXB13 expression, which subsequently stabilizes HIF‐1α through protein–protein interactions, activates the HIF‐1 signaling pathway, and drives lipid metabolism reprogramming, ultimately promoting sorafenib resistance in HCC cells. Our findings reveal a novel lactylation‐mediated mechanism linking lipid metabolism reprogramming to sorafenib resistance in HCC, providing a robust theoretical foundation and promising targets for developing combination therapies aimed at overcoming sorafenib resistance and improving patient prognosis.

## AUTHOR CONTRIBUTIONS

Qingqing Xie and Fangxia Teng conceived this study and participated in the design of the methods, experimental validation, and drafting of the manuscript. Ting Ding and Huaizhe Zhang were responsible for data acquisition and analysis. Jian Huang and Shu Zhang were responsible for supervision, manuscript review, funding acquisition, and project management. All authors have reviewed and agreed to the current version of the manuscript. All authors participated in writing the article and approved the submitted version.

## CONFLICT OF INTEREST STATEMENT

The authors declare no conflicts of interest.

## ETHICS STATEMENT

Not applicable.

## Supporting information

Supporting Information S1

Figures S1–S7

Tables S1–S5

## Data Availability

The datasets analyzed during this study are available in the following repositories: the Cancer Genome Atlas (https://www.cancer.gov/ccg/research/genome‐sequencing/tcga) and Gene Expression Omnibus (https://www.ncbi.nlm.nih.gov/geo/). All other data are included in the article and its Supporting Information.
