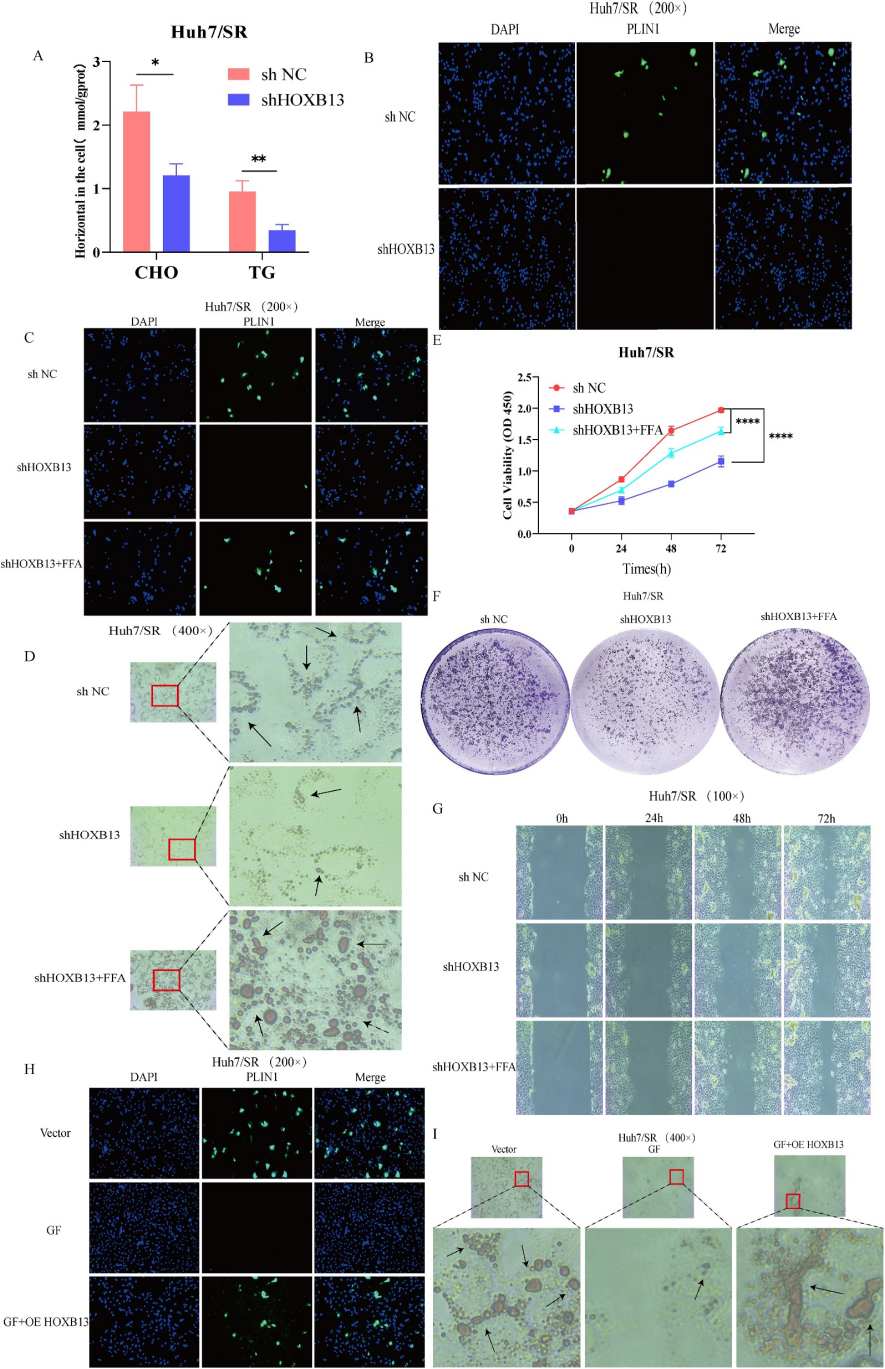# Correction to “Homeobox B13 activates the hypoxia‐inducible factor 1 pathway through histone lactylation thereby reprogramming lipid metabolism and promoting sorafenib resistance in hepatocellular carcinoma”

**DOI:** 10.1002/ccs3.70095

**Published:** 2026-08-18

Xie, Qingqing, Fangxia Teng, Ting Ding, Huaizhe Zhang, Jian Huang, and Shu Zhang. 2026. “Homeobox B13 activates the hypoxia‐inducible factor 1 pathway through histone lactylation thereby reprogramming lipid metabolism and promoting sorafenib resistance in hepatocellular carcinoma,” *Journal of Cell Communication and Signaling*: e70077. https://doi.org/10.1002/ccs3.70077.

In the originally published article, in Figure 5F, the HOXB13 western blot band representing Huh7/SR cells was inadvertently used to display the results for HCCLM3/SR cells. This error does not affect any scientific conclusions of the article.

Additionally, in Figure 6C–G, the negative control for the knockdown experiment was incorrectly labeled as “Vector.” The correct label should be “sh NC.” This does not affect the conclusions of the article.

We apologize for this error.

**FIGURE 5:**
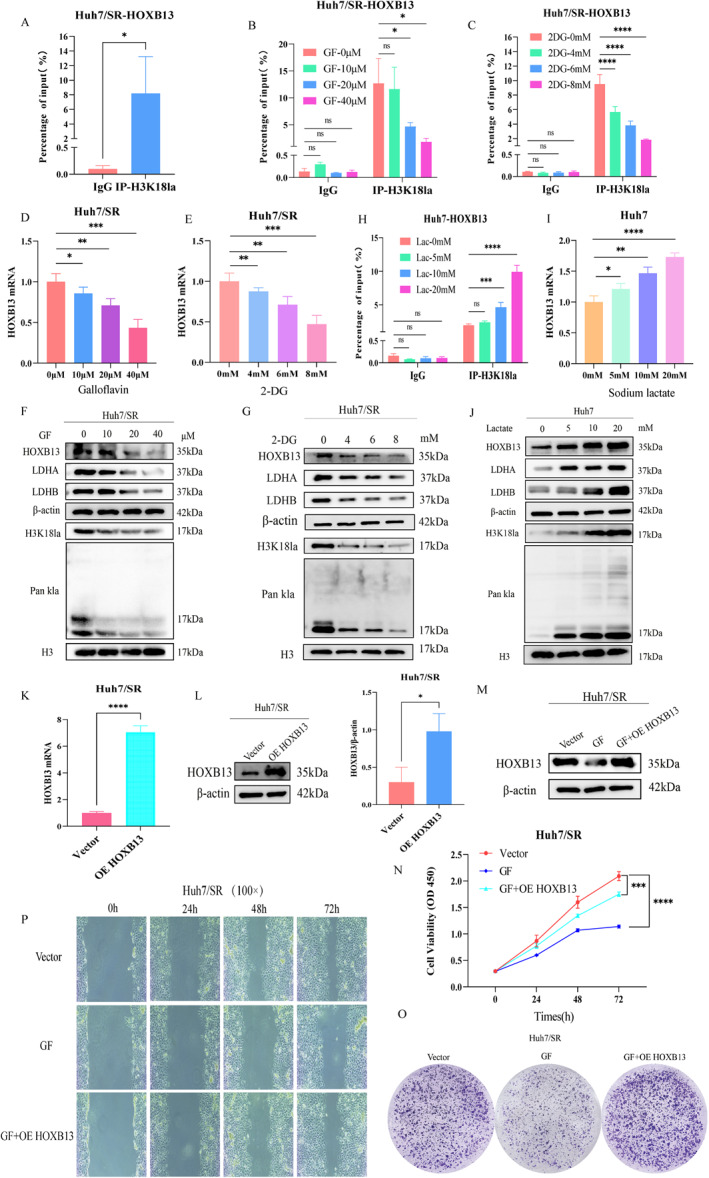


**FIGURE 6:**