# Supplementary material for: Homeobox B13 activates the hypoxia‐inducible factor 1 pathway through histone lactylation thereby reprogramming lipid metabolism and promoting sorafenib resistance in hepatocellular carcinoma
Source: J Cell Commun Signal. 2026 May 6;20(2):e70077. doi: 10.1002/ccs3.70077 (PMC13148139; doi:10.1002/ccs3.70077)
Supplement: Supplementary file 1 — Supporting Information S1 [file CCS3-20-e70077-s003.docx]

**Homeobox B13 activates the hypoxia-inducible factor-1 pathway through histone lactylation thereby reprogramming lipid metabolism and promoting sorafenib resistance in hepatocellular carcinoma**

Qingqing Xie^1,2#^, Fangxia Teng^1,2#^, Ting Ding^1,2^, Huaizhe Zhang^1,2^, Jian Huang^1,2^*, Shu Zhang^1,2^*

**1. Summary of Drug Duration and Concentration**

1.1 Drug Treatment Methodology

Sorafenib (HY-10201, MCE, USA) was dissolved in dimethyl sulfoxide (DMSO) to prepare a 10 mg/mL stock solution. GF (HY-W040118, MCE, USA) was dissolved in DMSO to prepare a 10 mM stock solution. 2-DG (HY-13966, MCE, USA) was prepared as a 1 M stock solution. Simvastatin (HY-17502, MCE, USA) was dissolved in DMSO to prepare a 1 mM stock solution. LW6 (HY-13671, MCE, USA) was prepared as a 0.5 mM stock solution. The aforementioned drugs were aliquoted into 200 μL portions, sealed with parafilm, and stored at –20°C in the dark, with long-term storage at –80°C in the dark. Sodium lactate (HY-B2227B, MCE, USA) was prepared as a 5.34 M stock solution. FFA (KC006, Xi'an Kunchuang, China) was used as stock solutions of 12 mM sodium oleate and 6 mM sodium palmitate. Before each experiment, the stock solutions were diluted with cell culture medium to the indicated concentrations. The final concentration of DMSO in all treatment groups did not exceed 0.1% (v/v). To maintain stable drug concentrations, the culture medium containing the drugs was replaced every 12 hours (unless otherwise indicated).

Cell cloning assay: Cells were plated and cultured for 48 h, followed by treatment with complete medium containing the indicated drugs for 72 h. After washing with PBS, the medium was replaced with drug-free complete medium, and cells were cultured for an additional 10–14 days.

Wound healing assay: Cells were pretreated with the indicated drugs for 24 h. Following wounding, the cells were cultured continuously in serum-free medium containing the drugs for the remainder of the experiment.

CCK-8 assay: Cells were plated and allowed to adhere. For inhibitory drugs, the cells were treated for 48 h when they reached approximately 80% confluence before detection. For sodium lactate, the cells were treated for 48 h when they reached approximately 60% confluence before detection.

Lipid metabolism assay: Cells were plated and allowed to adhere. For drugs inhibiting lipid accumulation, the cells were treated for 48 h when they reached approximately 80% confluence before detection. For sodium lactate and FFA treatments, cells were treated for 48 h upon reaching approximately 60% confluence before detection.

Immunofluorescence assay: Cells were plated and allowed to adhere. For inhibitory drugs, the cells were treated for 48 h when they reached approximately 60% confluence before detection. For sodium lactate treatment, cells were treated for 48 h upon reaching approximately 30% confluence before detection.

1.2 Drug Treatment Concentrations

Simvastatin: 40 μM for Huh7/SR cells, 50 μM for HCCLM3/SR cells.

LW6: 16 μM for Huh7/SR cells, 40 μM for HCCLM3/SR cells.

Rescue experiment: GF + OE HOXB13 (GF: 20 μM).

FFA: 0.5 mmol/L (300 μM sodium oleate, 200 μM sodium palmitate).

Drug combinations: GF (20 μM for Huh7/SR, 40 μM for HCCLM3/SR) + Simvastatin (40 μM); 2-DG (6 mM for Huh7/SR, 8 mM for HCCLM3/SR) + Simvastatin (50 μM).

**2. Western blot antibody information**

H3 (PTM-1002RM, PTM Bio, Dilution ratio 1:8,000), H3K18la (PTM-1406RM, PTM Bio, Dilution ratio 1:7,000), H3K23la (PTM-1413RM, PTM Bio, Dilution ratio 1:7,000), H4K5la (PTM-1409, PTM Bio, Dilution ratio 1:7,000), H4K12la (PTM-1411RM, PTM Bio, Dilution ratio 1:7,000), β-actin (66009-1-Ig, Proteintech, Dilution ratio 1:10,000), HOXB13 (26384-1-AP, Proteintech, Dilution ratio 1:2,000), HIF-1α (20960-1-AP, Proteintech, Dilution ratio 1:7,000), HIF-1β (84650-4-RR, Proteintech, Dilution ratio 1:8,000), LDHA (84198-2-RR, Proteintech, Dilution ratio 1:10,000), LDHB (81963-1-RR, Proteintech, Dilution ratio 1:10,000).

**3. Immunofluorescence antibody information**

Information on the primary antibody: PLIN1 (27716-1-AP, Proteintech, Dilution ratio 1:500), HOXB13 (26384-1-AP, Proteintech, Dilution ratio 1:200), HIF-1α (66730-1-Ig, Proteintech, Dilution ratio 1:200).

Secondary antibody information: CoraLite@ Plus 488 antibody IgG (H+L) (srbAF488-1, Proteintech, 1:500), CoraLite@ Plus 594 antibody IgG (H+L) (RGAM004, Proteintech, 1:500).
