# Supplementary material for: Homeobox B13 activates the hypoxia‐inducible factor 1 pathway through histone lactylation thereby reprogramming lipid metabolism and promoting sorafenib resistance in hepatocellular carcinoma
Source: J Cell Commun Signal. 2026 May 6;20(2):e70077. doi: 10.1002/ccs3.70077 (PMC13148139; doi:10.1002/ccs3.70077)
Supplement: Supplementary file 2 — Figures S1–S7 [file CCS3-20-e70077-s002.docx]

**Homeobox B13 activates the hypoxia-inducible factor-1 pathway through histone lactylation thereby reprogramming lipid metabolism and promoting sorafenib resistance in hepatocellular carcinoma**

Qingqing Xie^#^, Fangxia Teng^#^, Ting Ding, Huaizhe Zhang Jian Huang*, Shu Zhang*

**Supplementary Figure**


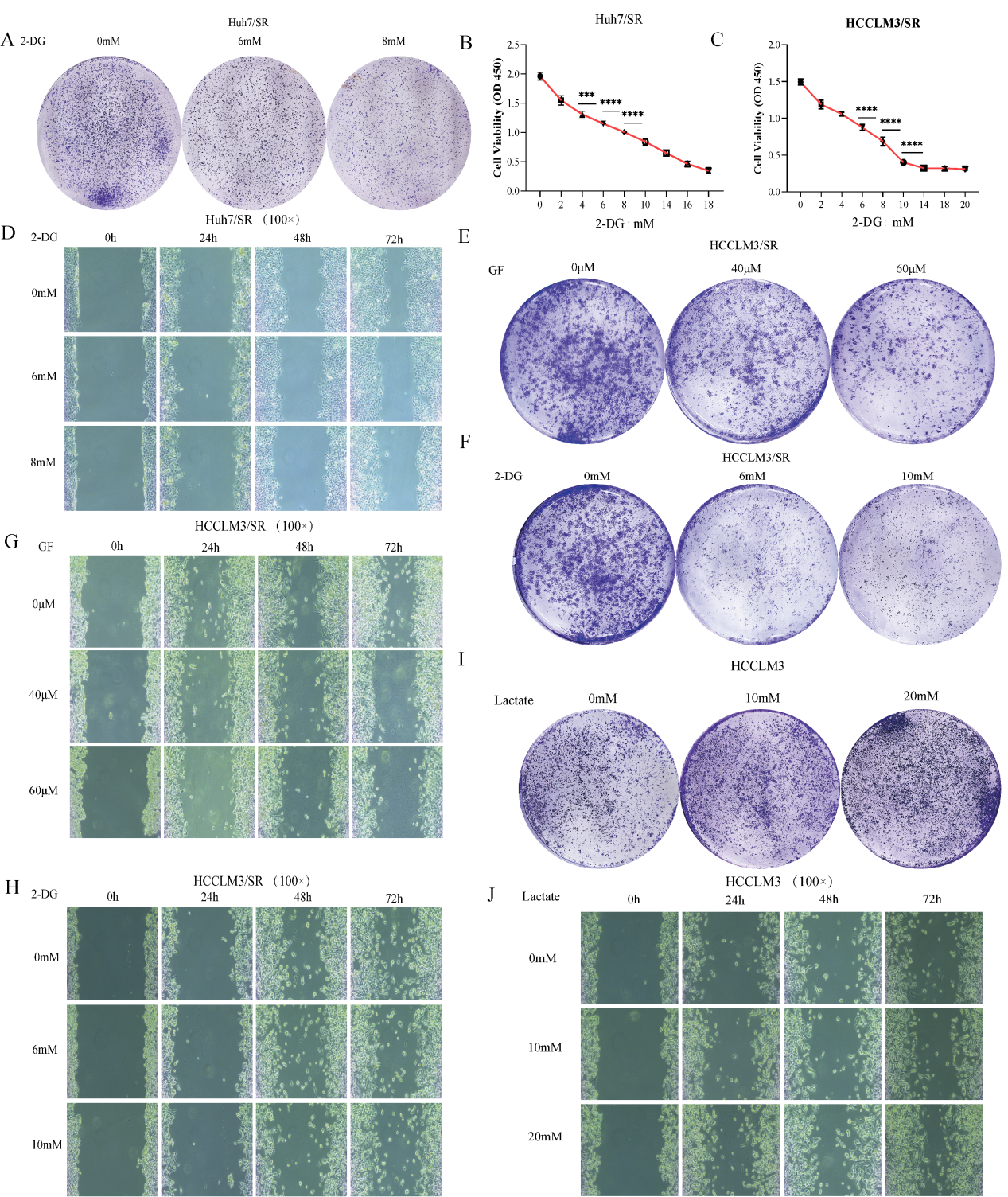


Supplementary Figure 1. Histone Kla influences sorafenib resistance in HCC

(A-H) Effects of 2-DG or GF treatment on cell proliferation and migration. (I-J) Effects of sodium lactate treatment on cell proliferation and migration. n=3 (independent biological replicates), ****P* <0.001, *****P* <0.0001.


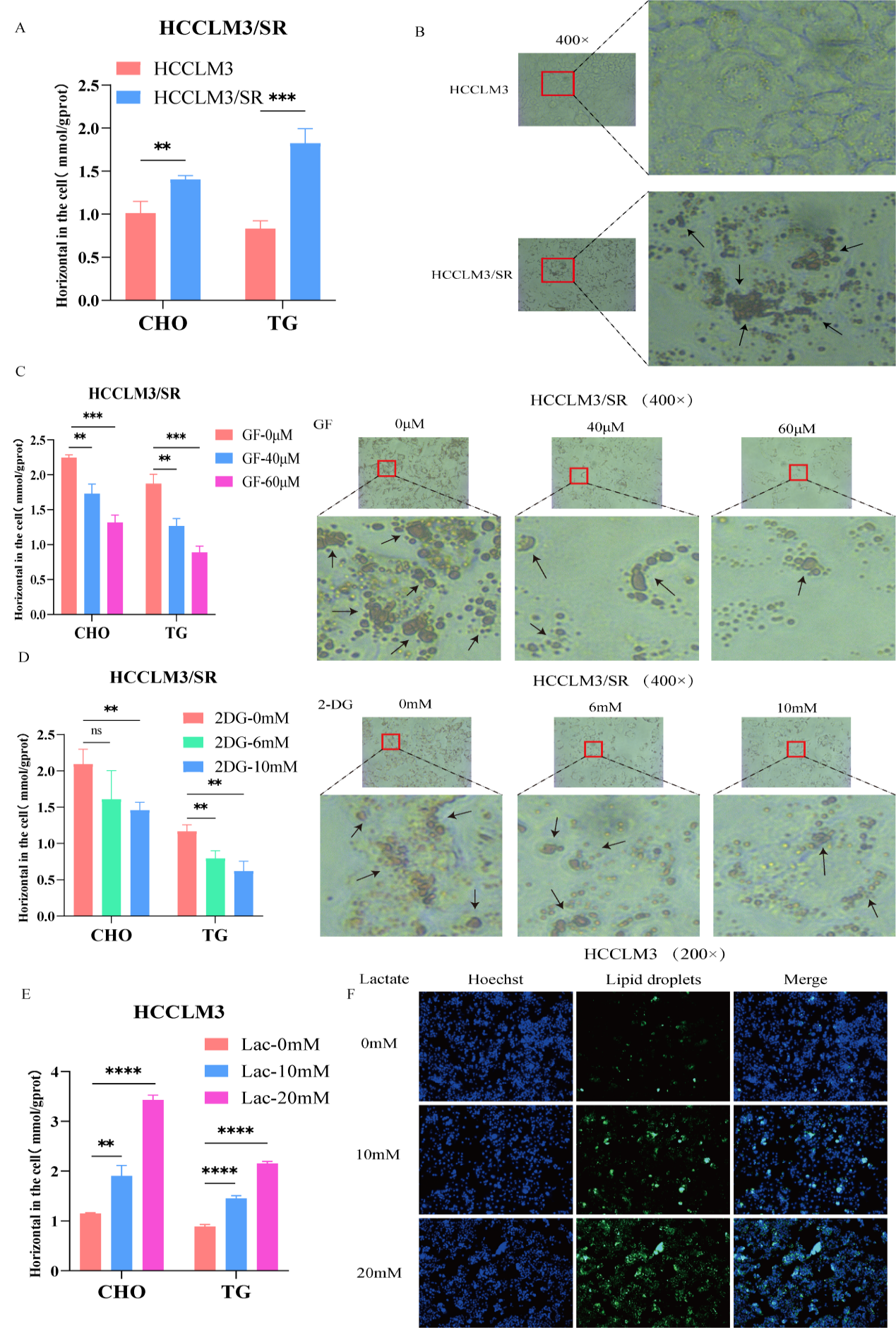


Supplementary Figure 2. Histone Kla drives lipid metabolic reprogramming

(A-B) Differential analysis of CHO, TG, and LD levels between sensitive and resistant cells. (C-D) Effects of GF or 2-DG treatment on lipid metabolism. (E-F) Effects of sodium lactate treatment on lipid metabolism. n=3 (independent biological replicates, results expressed as mean ± SD). ns: no statistical significance, ** *P* <0.01, *** *P* <0.001, **** *P* <0.0001.


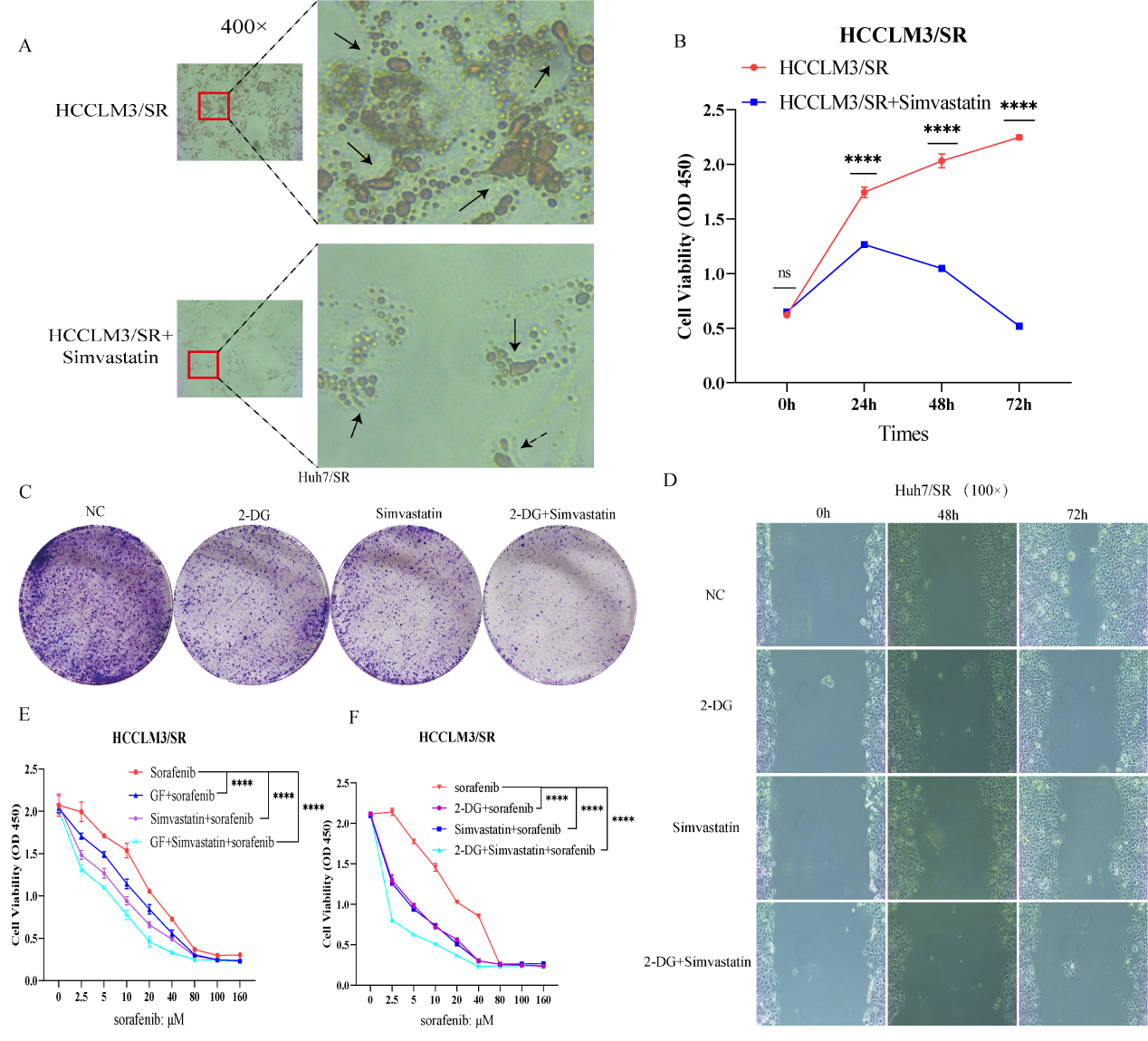


Supplementary Figure 3. Histone Kla-driven lipid metabolism reprogramming affects sorafenib resistance in HCC

(A) Effect of simvastatin treatment on LD accumulation. (B) Effect of simvastatin treatment on cell proliferation. (C-D) Effects of simvastatin combined with GF or 2-DG on cell proliferation and migration. (E-F) Changes in solanofenib sensitivity of cells after co-administration of simvastatin with GF or 2-DG. n=3 (independent biological replicates). ns: not statistically significant, **** *P* <0.0001.


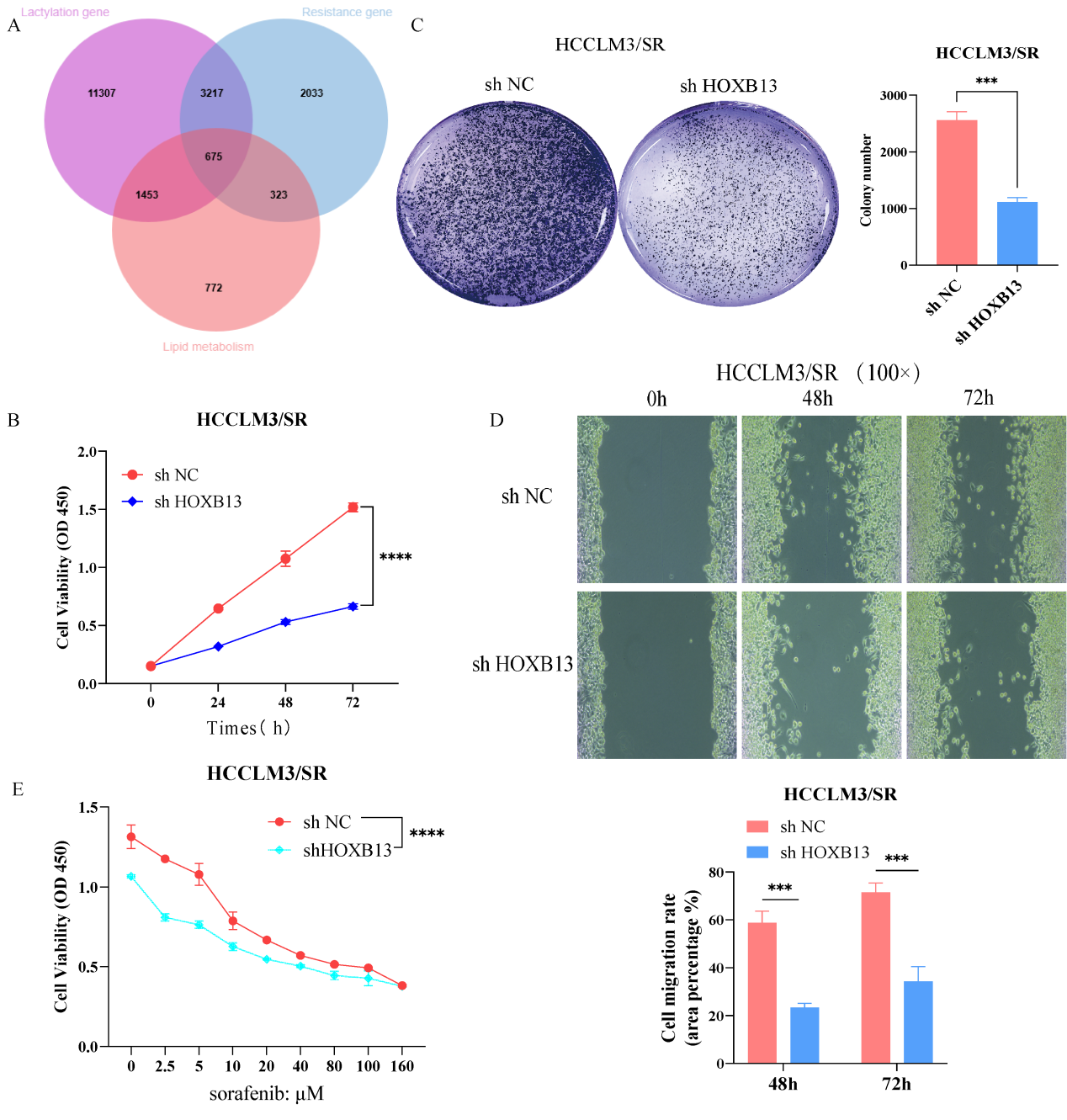


Supplementary Figure 4. HOXB13 is a key effector molecule in histone Kla-driven lipid metabolism reprogramming

(A) Veen diagram analysis of H3K18la-modified target molecules, lipid metabolism-related genes, and sorafenib resistance differential genes in HCC. (B-C) Effects of HOXB13 knockdown on cell proliferation. (D) Effects of HOXB13 knockdown on cell migration. (E) Changes in sorafenib treatment sensitivity in HOXB13 knockdown cells. n=3 (independent biological replicates, results expressed as mean ± SD). ****P* <0.001, *****P* <0.0001.


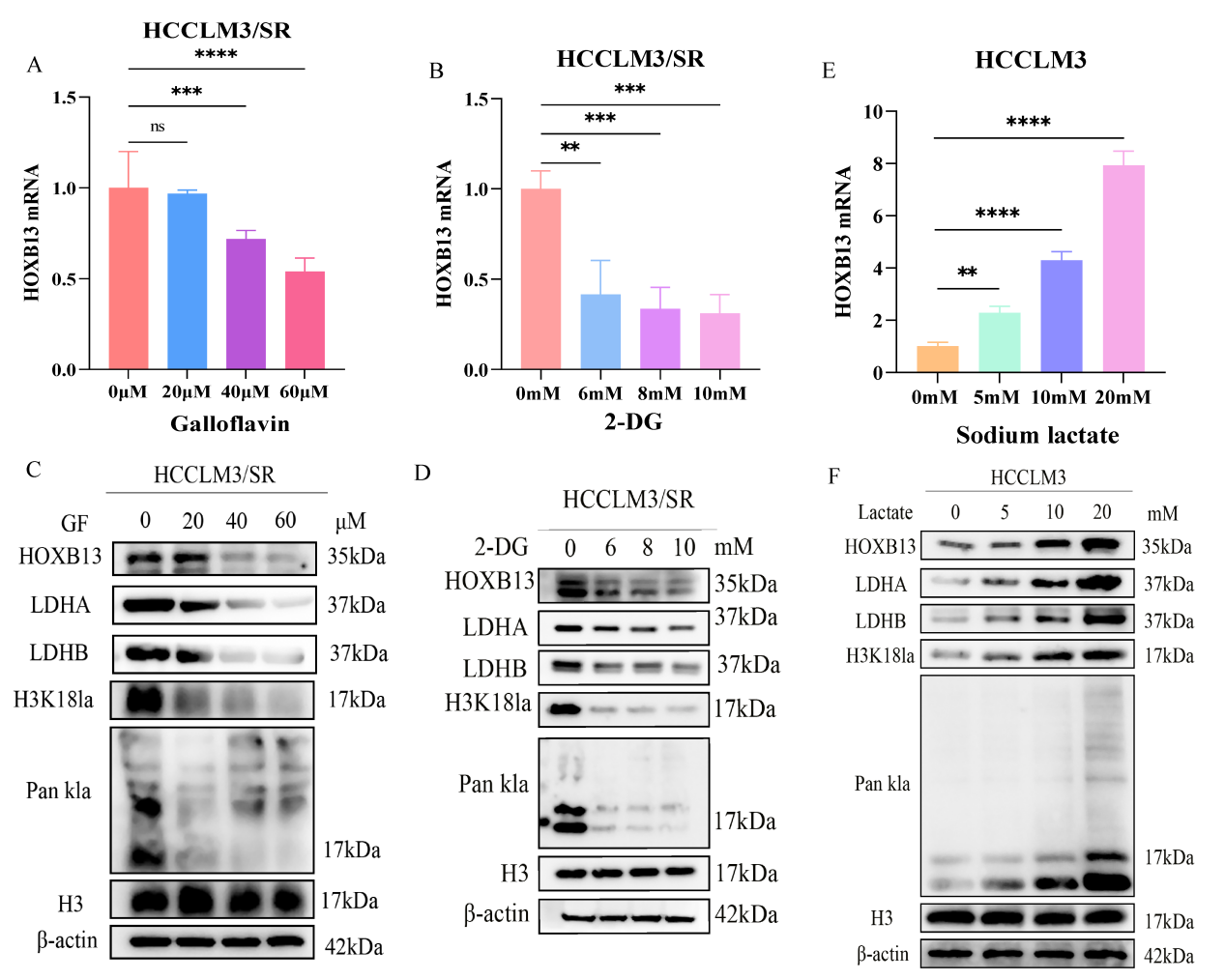


Supplementary Figure 5. Histone Kla regulates transcriptional activation of HOXB13 (A-D) Effects of 2-DG or GF treatment on HOXB13 expression. (E-F) Effects of sodium lactate treatment on HOXB13 expression. n=3 (independent biological replicates, results expressed as mean ± SD; representative bands shown by Western blot). ns: no statistical significance, ** *P* <0.01, *** *P* <0.001, **** *P* <0.0001.


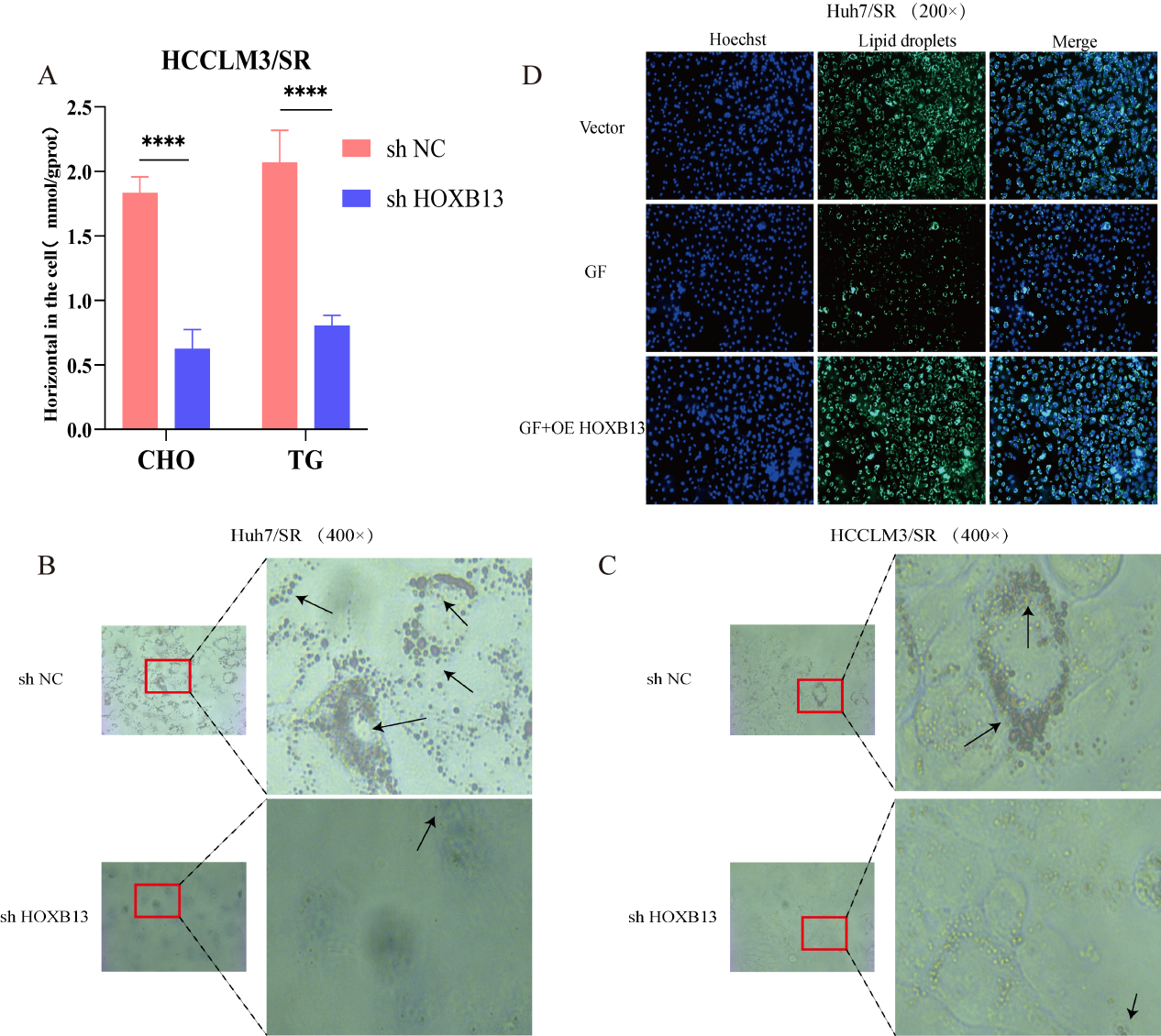


Supplementary Figure 6. Effects of HOXB13 knockdown on lipid metabolism

(A) Effects of HOXB13 knockdown on cell CHO and TG levels. (B-C) Effects of HOXB13 knockdown on cellular LD accumulation. (D) Effect of OE HOXB13 on LD levels after GF treatment. n=3 (independent biological replicates, results expressed as mean ± SD). **** *P* <0.0001.


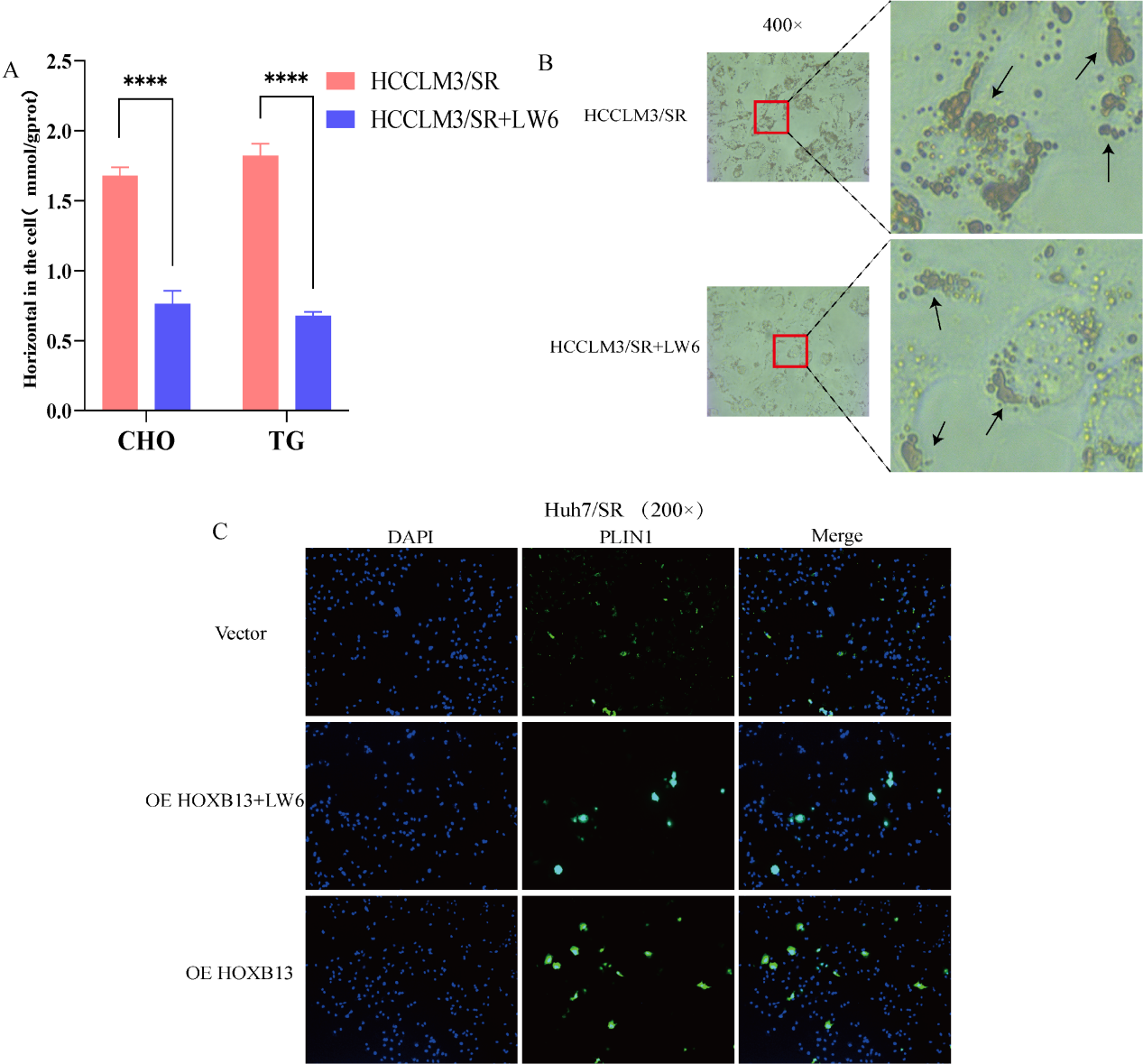


Supplementary Figure 7. The H3K18la/HOXB13/HIF-1 axis reprograms lipid metabolism in HCC resistant cells

(A-B) Effects of HIF-1 signaling pathway inhibition on lipid metabolism. (C) Effects of LW6 treatment after OE HOXB13 on lipid metabolism. n=3 (independent biological replicates, results expressed as mean ± SD).**** *P* < 0.0001.
